# Disruption of Protein Mannosylation Affects *Candida guilliermondii* Cell Wall, Immune Sensing, and Virulence

**DOI:** 10.3389/fmicb.2016.01951

**Published:** 2016-12-02

**Authors:** María J. Navarro-Arias, Tatiana A. Defosse, Karine Dementhon, Katalin Csonka, Erika Mellado-Mojica, Aline Dias Valério, Roberto J. González-Hernández, Vincent Courdavault, Marc Clastre, Nahúm V. Hernández, Luis A. Pérez-García, Dhirendra K. Singh, Csaba Vizler, Attila Gácser, Ricardo S. Almeida, Thierry Noël, Mercedes G. López, Nicolas Papon, Héctor M. Mora-Montes

**Affiliations:** ^1^División de Ciencias Naturales y Exactas, Departamento de Biología, Universidad de GuanajuatoGuanajuato, Mexico; ^2^Biomolécules et Biotechnologies Végétales, Université François-Rabelais de ToursTours, France; ^3^Groupe d'Etude des Interactions Hôte-Pathogène, Université d'AngersAngers, France; ^4^Laboratoire de Microbiologie Fondamentale et Pathogénicité, Université Bordeaux 2, UMR-Centre National de la Recherche Scientifique 5234Bordeaux, France; ^5^Department of Microbiology, University of SzegedSzeged, Hungary; ^6^Centro de Investigaciones y de Estudios Avanzados del Instituto Politécnico Nacional (IPN)Guanajuato, Mexico; ^7^Departamento de Microbiologia, Centro de Ciências Biológicas, Universidade Estadual de LondrinaLondrina, Brazil; ^8^Institute of Biochemistry, Biological Research Center of the Hungarian Academy of SciencesSzeged, Hungary

**Keywords:** cell wall, mannosylation pathway, *Candida guilliermondii*, host-fungus interplay, virulence, protein glycosylation

## Abstract

The fungal cell wall contains glycoproteins that interact with the host immune system. In the prominent pathogenic yeast *Candida albicans*, Pmr1 acts as a Golgi-resident ion pump that provides cofactors to mannosyltransferases, regulating the synthesis of mannans attached to glycoproteins. To gain insight into a putative conservation of such a crucial process within opportunistic yeasts, we were particularly interested in studying the role of the *PMR1* homolog in a low-virulent species that rarely causes candidiasis, *Candida guilliermondii*. We disrupted *C. guilliermondii PMR1* and found that loss of Pmr1 affected cell growth and morphology, biofilm formation, susceptibility to cell wall perturbing agents, mannan levels, and the wall composition and organization. Despite the significant increment in the amount of β1,3-glucan exposed at the wall surface, this positively influenced only the ability of the mutant to stimulate IL-10 production by human monocytes, suggesting that recognition of both mannan and β1,3-glucan, is required to stimulate strong levels of pro-inflammatory cytokines. Accordingly, our results indicate *C. guilliermondii* sensing by monocytes was critically dependent on the recognition of *N*-linked mannans and β1,3-glucan, as reported in other *Candida* species. In addition, chemical remotion of cell wall *O*-linked mannans was found to positively influence the recognition of *C. guilliermondii* by human monocytes, suggesting that *O*-linked mannans mask other cell wall components from immune cells. This observation contrasts with that reported in *C. albicans*. Finally, mice infected with *C. guilliermondii pmr1*Δ null mutant cells had significantly lower fungal burdens compared to animals challenged with the parental strain. Accordingly, the null mutant showed inability to kill larvae in the *Galleria mellonella* infection model. This study thus demonstrates that mannans are relevant for the *C. guilliermondii*-host interaction, with an atypical role for *O*-linked mannans.

## Introduction

Several members of the *Candida* genus cause both superficial and systemic candidiasis, the latter resulting in significant morbidity and mortality, especially in immunosuppressed patients (Brown et al., 2012). Indeed, invasive candidiasis is ranked as the second most lethal infection caused by opportunistic fungal pathogens, and *Candida albicans* remains the most frequent species isolated from affected patients (Brown et al., 2012). However, other species of this genus can cause life-threatening infections and are regarded as emerging pathogens. *Candida guilliermondii* is an opportunistic yeast that accounts for 1–3% of all candidemia cases, most frequently in patients with oncological diseases (Girmenia et al., 2006; Pfaller et al., 2006; Savini et al., 2011). Although this organism is medically relevant, it is still considered a low-virulence species (Savini et al., 2011); therefore, its study can provide insights into differences in pathogenicity mechanisms, virulence and interaction with host cells from that of other more virulent species, such as *C. albicans*.

Host immune sensing of fungal antigens is a key step in the development of a protective anti-fungal response, and recognition of fungal cell wall constituents initiates this process (Netea et al., 2008). Thus far, the *C. albicans* cell wall is the most well studied fungal structure, and models about its composition, structure, organization and relevance during the interaction with host cells are available (Klis et al., 2001; Díaz-Jiménez et al., 2012; Gow and Hube, 2012; Gow et al., 2012; Netea et al., 2015). The *C. albicans* cell wall is composed of structural polysaccharides (chitin, β1,3- and β1,6-glucans) that surround the plasma membrane (inner wall layer) and an outer layer composed of *N*- and *O*-linked mannoproteins (Klis et al., 2001). All these components are recognized as pathogen-associated molecular patterns and are capable of stimulating both cytokine production and phagocytosis by innate immune cells (Martínez-Álvarez et al., 2014). Several receptors on the surface of immune cells involved in the sensing of *C. albicans* cell wall components have been identified. For example, the *N*-linked mannans are recognized by mannose receptor, DC-SIGN, Mincle, dectin-2 and dectin-3 (Netea et al., 2006; Cambi et al., 2008; Wells et al., 2008; Saijo et al., 2010; Zhu et al., 2013), while *O*-linked mannans are sensed via TLR4 (Netea et al., 2006). β1,3-Glucan stimulates signaling pathways in the immune cells when interacting with dectin-1 and TLR2 (Brown and Gordon, 2001; Netea et al., 2006), and chitin has been recently reported to be sensed by the mannose receptor and the intracellular molecules TLR9 and NOD2 (Wagener et al., 2014). Despite this significant advance in understanding the *C. albicans*-immune system interactions, little is known about *C. guilliermondii* immune sensing. Thus far, it has been established that *C. guilliermondii* is a moderate stimulus for production of granulocyte-macrophage colony-stimulating factor and the complement components C3 and factor B by human monocytes (Høgåsen et al., 1995). Accordingly, *C. guilliermondii* displayed a limited capacity to stimulate tumor necrosis factor α (TNFα) when co-incubated with peritoneal macrophages (Aybay and Imir, 1996). However, it is readily phagocytosed by murine polymorphonuclear cells, bone marrow cells, peritoneal macrophages and spleen cells, when compared to the phagocytic index of *C. albicans* cells (Vecchiarelli et al., 1985).

Similar to the studies dealing with the immune sensing, the *C. guilliermondii* cell wall structure and composition have been poorly studied, but its cell wall is nevertheless assumed to be similar to the one described in *C. albicans*. *C. guilliermondii* cell wall contains chitin, which increases in amount in response to exposure to sub-lethal concentrations of caspofungin (Walker et al., 2013). Furthermore, structural analysis of *C. guilliermondii* cell wall mannans indicated the presence of α1,2-, α1,3- and β1,2-mannose units, suggesting a similar organization to the *C. albicans* mannans (Okawa et al., 2006).

Mannan relevance for *Candida* spp. cell wall integrity, virulence, and sensing by innate immune cells has been mainly assessed using mutant cells lacking specific enzymes with key roles in the assembly of either *N*- or *O*-linked mannans (Bates et al., 2005, 2006, 2013; Munro et al., 2005; Prill et al., 2005; Mora-Montes et al., 2007, 2010; Hall et al., 2013; West et al., 2013; Pérez-García et al., 2016). Pmr1 is a Golgi-resident P-type ATPase ion pump that provides the cofactor Mn^2+^ to mannosyltransferases involved in both *N*-linked and *O*-linked glycosylation (Bates et al., 2005). A previously generated *C. albicans pmr1*Δ null mutant displayed a strong defect in the cell wall composition and elaboration of both *N*-linked and *O*-linked mannans (Bates et al., 2005); and thus, produced a poor cytokine response in human peripheral-blood mononuclear cells (PBMCs) and dendritic cells, and its phagocytosis by macrophages was significantly affected (Netea et al., 2006; Cambi et al., 2008; McKenzie et al., 2010); these findings underscore the relevance of both *N*- and *O*-linked mannans for a proper *C. albicans*-immune cell interaction. Furthermore, functional differences arising from β-elimination by trimming of *O*-linked mannans or removal of *N*-linked mannans upon treatment with endoglycosidase H (endo H) have also added to our understanding of the physiological role of mannans in the biology of *Candida* spp. (Hamada et al., 1981; Hazen and Glee, 1994; Mormeneo et al., 1994; Goins and Cutler, 2000; Spreghini et al., 2003; Pérez-García et al., 2016).

Here, we disrupted *C. guilliermondii PMR1* (Cg*PMR1*) and found that the loss of proper protein mannosylation affected cell growth and morphology, biofilm formation, cell wall composition and organization, and interaction with human PBMCs and the cell line J774 of murine macrophages. In addition, virulence was affected in the model of systemic candidiasis in both the invertebrate *Galleria mellonella* and mice. Interestingly, we also found that *C. guilliermondii* and *C. albicans O*-linked mannans are similar in composition, but the former mask other cell wall components from interaction with human PBMCs, preventing a significant increase in cytokine production compared to that generated by the *pmr1*Δ null mutant.

## Results

### Identification of *C. guilliermondii PMR1* and construction of a null mutant strain

The Cg*PMR1* sequence was identified following a standard protein BLAST analysis at the NCBI website (http://www.ncbi.nlm.nih.gov/), using the protein sequence of *C. albicans* Pmr1 (GenBank accession code XP_720380) as query. The best hit was the hypothetical protein PGUG_00945 (GenBank accession code EDK36847), which is encoded by the locus CH408155 (GenBank accession code CH408155, region: 1663175.1665946). This open reading frame (ORF) spans 2772 bp and is predicted to encode a polypeptide of 923 amino acids, with 76 and 87% identity and similarity to *C. albicans* Pmr1, respectively. The putative protein is predicted to bear eight transmembrane domains and the canonical motif ^353^DKTGTLT, which contains the aspartic acid residue involved in the phosphorylation of P-type ATPases (Lutsenko and Kaplan, 1995; Bates et al., 2005). Furthermore, this ORF is unlikely to be the ortholog of other membrane-bound ATPases, such as *ENA2, PMC1*, or *PMA1*, as the identity and similarity to those proteins was relatively low (30.8 and 48.3%, 28.3 and 46.8%, 27.1 and 47.8%, respectively).

**Figure 1:**
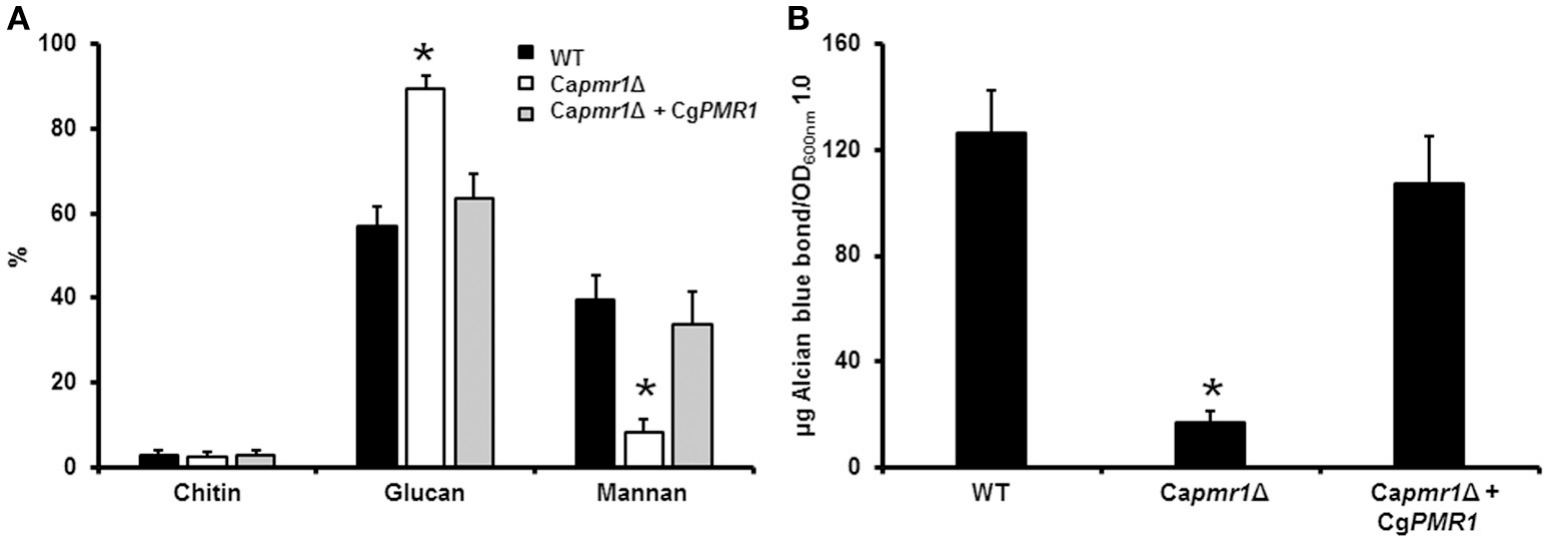
**Cg***PMR1*** is the functional ortholog of Ca***PMR1*****. Cg*PMR1* was expressed in the *C. albicans pmr1*Δ null mutant, as described in Experimental procedures, and the cell wall was isolated and analyzed by High-Performance Anion-Exchange Chromatography with Pulsed Amperometric Detection to assess the carbohydrate content **(A)**. In addition, the ability of cells to bind Alcian blue was analyzed **(B)**. ^*^P < 0.05. Strains used are: NGY152 (WT), Ca*pmr1*Δ (NGY355), Ca*pmr1*Δ + Cg*PMR1* (HMY186).

To demonstrate that Cg*PMR1* is the functional ortholog of the Ca*PMR1*, we complemented a *C. albicans pmr1*Δ null mutant (Bates et al., 2005) with *C. guilliermondii PMR1* under the control of its native promoter. The phenotypical analysis of the mutant strain indicated that *C. guilliermondii PMR1* restored the cell wall composition of the *C. albicans pmr1*Δ null mutant to levels similar to those found in the wild-type (WT) control cells (Figure 1A). Furthermore, the cell wall phosphomannan content was restored upon transformation with the *C. guilliermondii* gene (Figure 1B). Therefore, *C. guilliermondii PMR1* is the functional ortholog of *C. albicans PMR1*.

**Figure 2:**
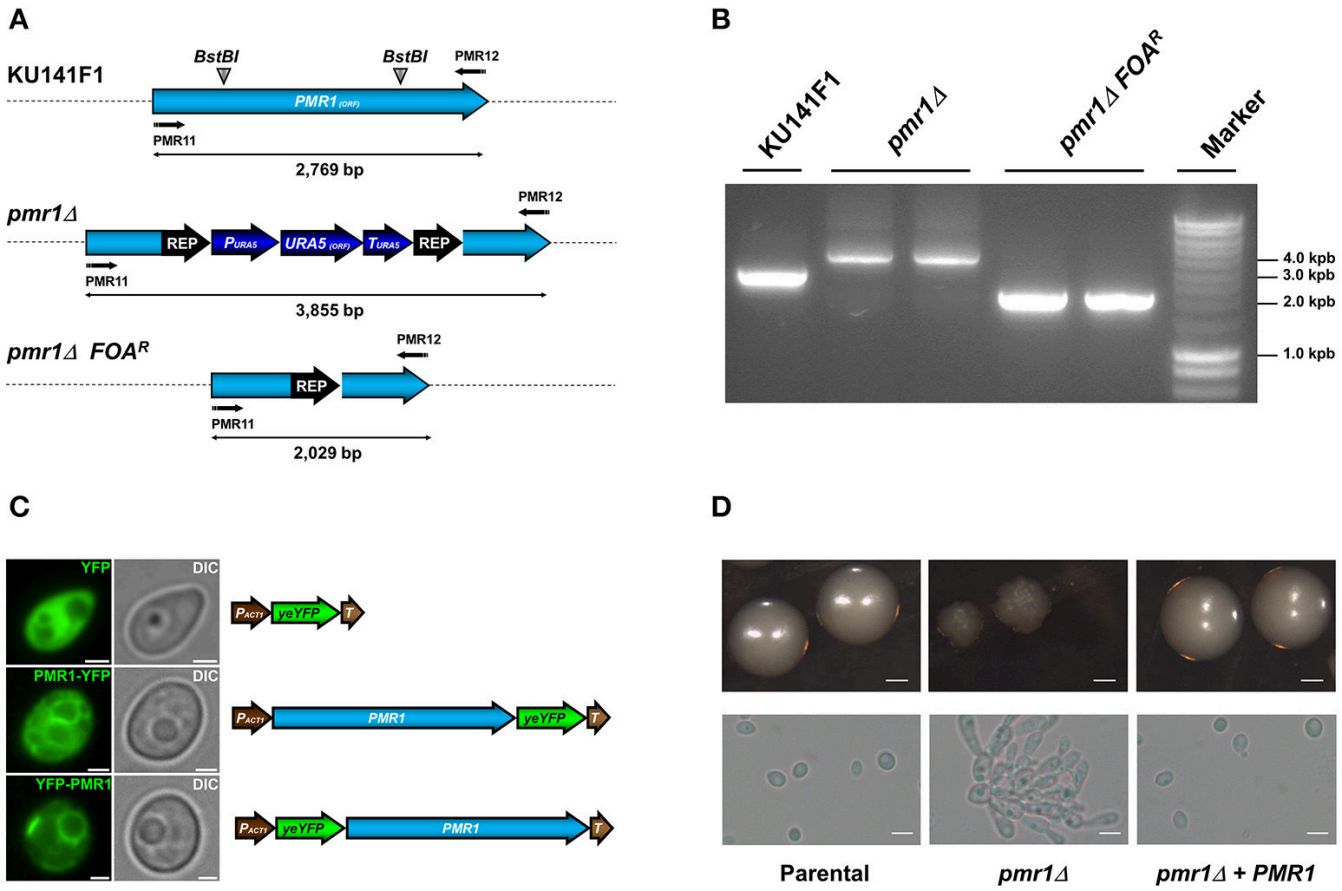
**Generation of the ***C. guilliermondii*** mutants used in this study and colony and cell morphology. (A)** Schematic representation of the wild type and disrupted *PMR1* loci. The *PMR1* was disrupted in the strain KU141F1 using the *URA5* blaster system. **(B)** Generation of the null mutant was confirmed by PCR reactions using the primer pair PMR11 5′-CTGAGAGGATCCATGACAGAGAACCCGTTCGATGTG-3′ and PMR12 5′-CTGAGAACTAGTTACGCTGTAGCTGACTCCATTCAT-3′ that amplifies the whole *PMR1* locus. All the generated amplicons displayed the expected sizes, as indicated in **(A)** of the figure. **(C)** Subcellular localization of YFP alone, Pmr1-YFP and YFP-Pmr1 fusion proteins in the *pmr1*Δ strain. Scale bar = 2 μm. **(D)** Colony and cell morphology of *C. guilliermondii* strains. Cells were grown on SC medium at 30°C for 36 h, scale bar = 1 mm. Alternatively, strains were cultured in SC broth at 30°C for 18 h; scale bar = 10 μm. The strains used are: KU141 (Parental), HMY134 (*pmr1*Δ) and HMY138 (*pmr1*Δ + *PMR1*).

The Cg*PMR1* disruption was performed in the KU141F1 recipient strain (genotype *ura5, ku70*) using the *URA5* blaster strategy (Foureau et al., 2013b). Replacement of the *PMR1* ORF with the disruption cassette was confirmed by PCR (Figures 2A,B). To generate a re-integrant control strain, the *C. guilliermondii pmr1*Δ null mutant was subjected to recycling of the *URA5* blaster cassette, yielding a Ura^−^ strain (*pmr1*Δ FOA^R^, Figures 2A,B), and transformed either with pG-URA5-*PMR1*-YFP or pG-URA5-YFP-*PMR1*. These constructions allow the expression of chimeric proteins Pmr1-YFP or YFP-Pmr1. When observed by epifluorescence microscopy, both series of transformed cells exhibited a network-shaped fluorescent signal, suggesting the targeting of the Pmr1-YFP and YFP-Pmr1 fusion proteins in the native organelle where Pmr1 was previously reported in other yeast species (Huh et al., 2003), i.e., the Golgi complex (Figure 2C). A representative *C. guilliermondii pmr1*Δ null mutant strain as well as a representative *pmr1*Δ FOA^R^ + pG-URA5-*PMR1*-YFP re-integrant strain (abbreviated *pmr1*Δ + *PMR1*) were selected for further phenotype analysis.

### Colony, cell morphology and ability to form biofilms of the *C. guilliermondii pmr1*Δ null mutant

The null mutant tended to grow as small colonies with irregular border and surface. In addition, the mutant was found to spontaneously form pseudohyphae and cell aggregates when cultured in liquid media (Figure 2D). The cell aggregates were dispersed upon addition of 2 units/mL chitinase (Bates et al., 2006; Pérez-García et al., 2016), suggesting defects in cell separation associated to alterations of the cell wall (data not shown). The growth rate of the *C. guilliermondii pmr1*Δ null mutant was significantly reduced, with doubling times of 3.64 ± 0.35 h, when compared with the control strains: 2.64 ± 0.17 h, and 2.85 ± 0.21 h for the parental (KU141) and re-integrant (*pmr1*Δ + *PMR1*) strains, respectively (*P* < 0.05). Experiments conducted in the presence of 2 units/mL chitinase showed similar results (data not shown). The *pmr1*Δ null mutant, but not the parental or re-integrant control cells failed to grow in SC medium supplemented with 15 mM EGTA, suggesting a strong defect in calcium mobilization (data not shown). We next tested the ability of the *pmr1*Δ null mutant to form biofilms on a polystyrene surface. The *pmr1*Δ null mutant displayed a 41.7 ± 13.8% reduction in the ability to form biofilms, which was statistically significant when compared to the parental and re-integrant control strains (100 ± 14.1% and 97.8 ± 10.6%, respectively; *P* < 0.05) Therefore, loss of *PMR1* affects *C. guilliermondii* growth, morphology, and ability to form biofilms.

### The cell wall composition, mannosylation, organization and susceptibility to cell wall perturbing agents is affected in the *pmr1*Δ null mutant

**Figure 3:**
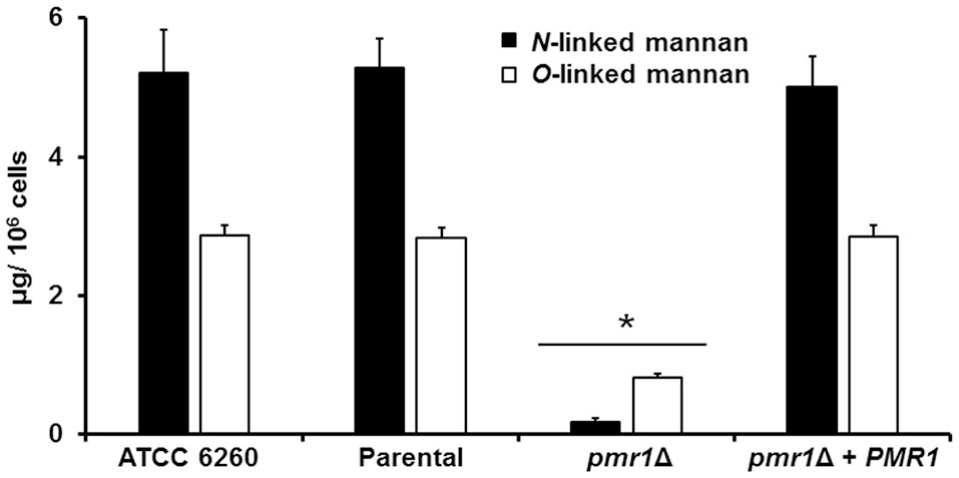
**The ***C. guilliermondii pmr1***Δ null mutant shows reduced levels of both ***N***-linked and ***O***-linked mannans at the cell wall**. The *C. guilliermondii* strains ATCC 6260, KU141 (Parental), HMY134 (*pmr1*Δ) and HMY138 (*pmr1*Δ + *PMR1*) were treated with endoglycosidase H or β-eliminated to remove *N*-linked mannans or *O*-linked mannans, respectively. The released material was concentrated and sugar content was quantified as described in Experimental procedures. Data are means ± SD of three independent experiments performed in duplicates. ^*^P < 0.05.

To decipher a putative role of *PMR1* on *C. guilliermondii* cell wall composition, yeast cell walls were isolated, acid hydrolyzed and analyzed by High-Performance Anion-Exchange Chromatography with Pulsed Amperometric Detection (HPAEC-PAD) as detailed in the Experimental procedures. Importantly, the parental strain (KU141) used for generating the *pmr1*Δ null mutant also bears a mutation in *KU70* (Foureau et al., 2013b). Therefore, to demonstrate the lack of effect of the *KU70* mutation on the studied phenotypes, we included in the analyses the ATCC 6260 reference strain (WT) as an additional control. The cell wall of control strains was similar in carbohydrate and protein contents, as well as in wall porosity to the polycation DEAE-dextran, an indirect reporter of mannan arrangement (Cheng et al., 2011; Pérez-García et al., 2016). The *pmr1*Δ null mutant displayed significant changes in wall composition, with a 5.8-fold and 1.8-fold increment in chitin and glucan levels, respectively (Table 1). These modifications were accompanied by a 13-fold reduction in mannan content, whereas phosphomannan was basically absent from the null mutant cell wall (Table 1). No significant differences in the *pmr1*Δ cell wall protein levels were found, but the cell wall porosity to DEAE-dextran was significantly reduced (Table 1). To confirm the defect in the cell wall mannan content, we removed either *N*-linked or *O*-linked mannans from the cell wall using endo H treatment or β-elimination, respectively, and quantified the amount of mannan released. The control strains did not display any significant difference in the content of both kinds of mannans, having about 65 and 35% of the mannan content released upon endo H treatment (*N*-linked mannans) and β-elimination (*O*-linked mannans), respectively (Figure 3). As expected, the content of both mannans in the *pmr1*Δ null mutant was significantly reduced, with a reduction of 97 and 71% in the levels of *N*-linked and *O*-linked mannans, respectively (Figure 3).

**Table 1:** **Cell wall analysis of Cg***pmr1***Δ null mutant and control strains**.

**Strain**	**Cell wall abundance**			**Phosphomannan content (μg)^a^**	**Porosity (%)^b^**	**Protein (μg)^c^**
	**Chitin (%)**	**Mannan (%)**	**Glucan (%)**			
ATCC 6260	1.8±1.0	50.8±0.7	47.4±2.5	121.9±12.4	61.4±6.5	144.2±24.1
KU141 (Parental)	1.9±0.6	49.2±2.6	48.9±2.2	119.5±12.8	59.7±3.6	139.6±26.5
HMY134 (*pmr1*Δ)	9.3±1.4^*^	3.9±2.6^*^	86.7±4.8^*^	0.17±15.0^*^	37.5±3.1^*^	151.1±20.8
HMY138 (*pmr1*Δ + *PMR1*)	1.2±1.0	53.1±2.2	45.7±3.1	115.6±10.7	63.0±6.1	141.8±19.1
KU141 (Parental)^d^	2.3±0.6	32.5±1.9^†^	65.2±1.1^†^	ND	ND	ND
HMY134 (*pmr1*Δ)^d^	8.7±1.4^*^	1.1±1.0	90.2±0.9	ND	ND	ND
HMY138 (*pmr1*Δ + *PMR1*)^d^	1.6±1.0	34.5±0.9^†^	63.9±2.2^†^	ND	ND	ND
KU141 (Parental)^e^	2.0±1.7	17.8±2.4^†^	80.2±3.3^†^	ND	ND	ND
HMY134 (*pmr1*Δ)^e^	10.3±1.1	0.2±0.4^†^	89.5±2.6	ND	ND	ND
HMY138 (*pmr1*Δ + *PMR1*)^e^	2.0±1.5	18.6±1.8^†^	79.4±2.3^†^	ND	ND	ND

a *μg of Alcian Blue bound/OD_600_ = 1*.

b *Relative to DEAE-Dextran*.

c *μg of protein/mg of cell wall*.

d *Upon β-elimination*.

e *Upon treatment with endoglycosidase H*.

* *P <0.05*.

† *P <0.05, when compared to untreated cells*.

*ND, Not determined*.

**Figure 4:**
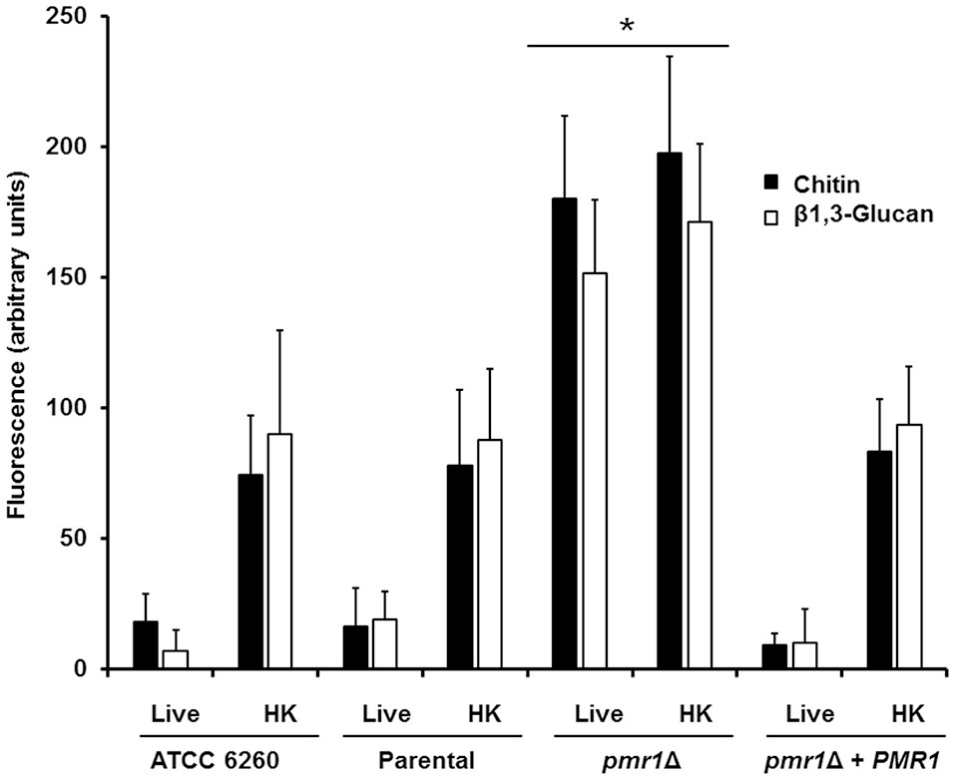
**The cell-wall structural polysaccharides, chitin and β1,3-glucan, are significantly exposed at the cell surface of the ***C. guilliermondii pmr1***Δ null mutant**. Live or heat-killed (HK) yeast cells were incubated with either fluorescein isothiocyanate-wheat germ agglutinin conjugate (closed bars, labels chitin) or IgG Fc-Dectin-1 chimera (open bars, labels β1,3-glucan) as described in the Experimental procedures, inspected under fluorescence microscopy, and the fluorescence associated to 100 individual cells recorded. The strains used are: ATCC 6260, KU141 (Parental), HMY134 (*pmr1*Δ) and HMY138 (*pmr1*Δ + *PMR1*) ^*^P < 0.05, when compared with live and HK cells from KU141, HMY134, and HMY138 strains.

Furthermore, we assessed whether loss of *PMR1* affected the organization of structural polysaccharides within the cell wall. For this purpose, we used a fluorescein isothiocyanate-wheat germ agglutinin conjugate (WGA-FITC) and an IgG Fc-Dectin-1 chimera, which are known to bind chitin and β1,3-glucan, respectively (Graham et al., 2006; Mora-Montes et al., 2011; Marakalala et al., 2013; Estrada-Mata et al., 2015), and analyzed their abilities to bind these polysaccharides. Results shown in Figure 4, Figures 1S, 2S indicate that live ATCC 6260, parental (KU141), and re-integrant control strains were minimally labeled by either WGA-FITC or IgG Fc-Dectin-1 chimera, indicating a small proportion of chitin or β1,3-glucan exposed at the surface of the cell wall. However, the live *pmr1*Δ null mutant was significantly bound by both lectins (Figure 4, Figures 1S, 2S). The increased labeling was not associated to the morphology change in the *pmr1*Δ null mutant, as pseudohyphae and yeast cells from the ATCC 6260 and parental (KU141) strains showed a similar ability to bind either WGA-FITC or IgG Fc-Dectin-1 (data not shown). It has been reported that heat-inactivated *C. albicans* and *Candida parapsilosis* cells expose β1,3-glucan and chitin at the cell wall surface (Gow et al., 2007; Mora-Montes et al., 2011; Estrada-Mata et al., 2015; Pérez-García et al., 2016); hence, as anticipated, there was an increased binding by both lectins in heat-killed (HK) control *C. guilliermondii* cells (Figure 4, Figures 1S, 2S). Interestingly, there was not an increment in the labeling by both lectins in the HK *pmr1*Δ null mutant when compared to live null mutant cells, suggesting that most of chitin and β1,3-glucan is already exposed at the cell surface (Figure 4, Figures 1S, 2S).

**Figure 5:**
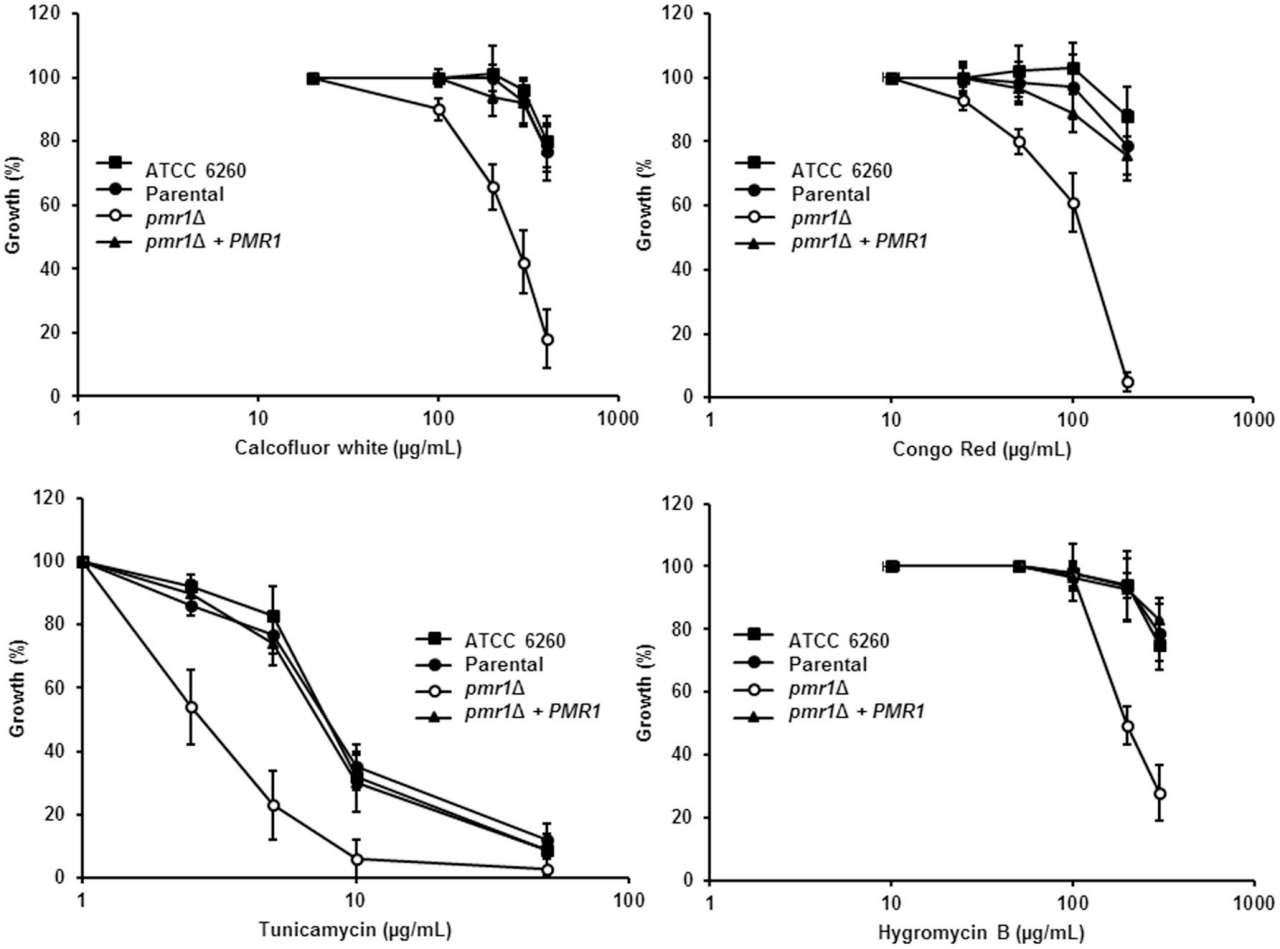
*****C. guilliermondii pmr1***Δ null mutant shows increased susceptibility to cell wall perturbing agents**. The *C. guilliermondii* strains ATCC 6260, KU141 (Parental), HMY134 (*pmr1*Δ), and HMY138 (*pmr1*Δ + *PMR1*) were incubated with different concentrations of either Calcofluor White, Congo Red, Tunicamycin, or Hygromycin B, and growth was determined after incubation for 24 h at 30°C. Growth data were normalized as percentage of those generated with the same strain without treatment. For data normalization, growth results are shown as percentage of those obtained with the same strain growing in the absence of any perturbing agent. Data are means ± SD of three independent experiments performed in duplicates. For all the agents tested, the null mutant sensitivity was significantly different to that of the control strains (*P* < 0.01 when compared by two-way ANOVA).

Next, we assessed the effect of cell wall perturbing agents and compounds associated with glycosylation defects on the growth of *C. guilliermondii* strains. The *pmr1*Δ null mutant showed increased susceptibility to Calcofluor white, Congo red, tunicamycin and hygromycin B (*P* < 0.01 in all cases; Figure 5). In addition, null mutant cells displayed an increased sensitivity to SDS, which affects the plasma membrane (Bates et al., 2006; Mora-Montes et al., 2007; Pérez-García et al., 2016) (data not shown). For all of the perturbing agents, the control strains were largely resistant, and no significant difference were found among them (Figure 5). Interestingly, the *pmr1*Δ null mutant displayed a reduced susceptibility toward fluconazole, but not to nystatin (not shown), with a fluconazole minimal inhibitory concentration (MIC) of 18.3 ± 3.8 μg/mL after 24 h incubation at 30°C (MIC for the ATCC 6260, parental (KU141) and re-integrant control strains are 7.1 ± 3.0 μg/mL, 8.5 ± 4.8 μg/mL, and 9.1 ± 3.3 μg/mL, respectively; *P* = 0.03). Therefore, the *pmr1*Δ null mutant has significant defects in cell wall composition, organization and fitness.

### Loss of *C. guilliermondii* protein mannosylation affects cytokine production by human PBMCs

**Figure 6:**
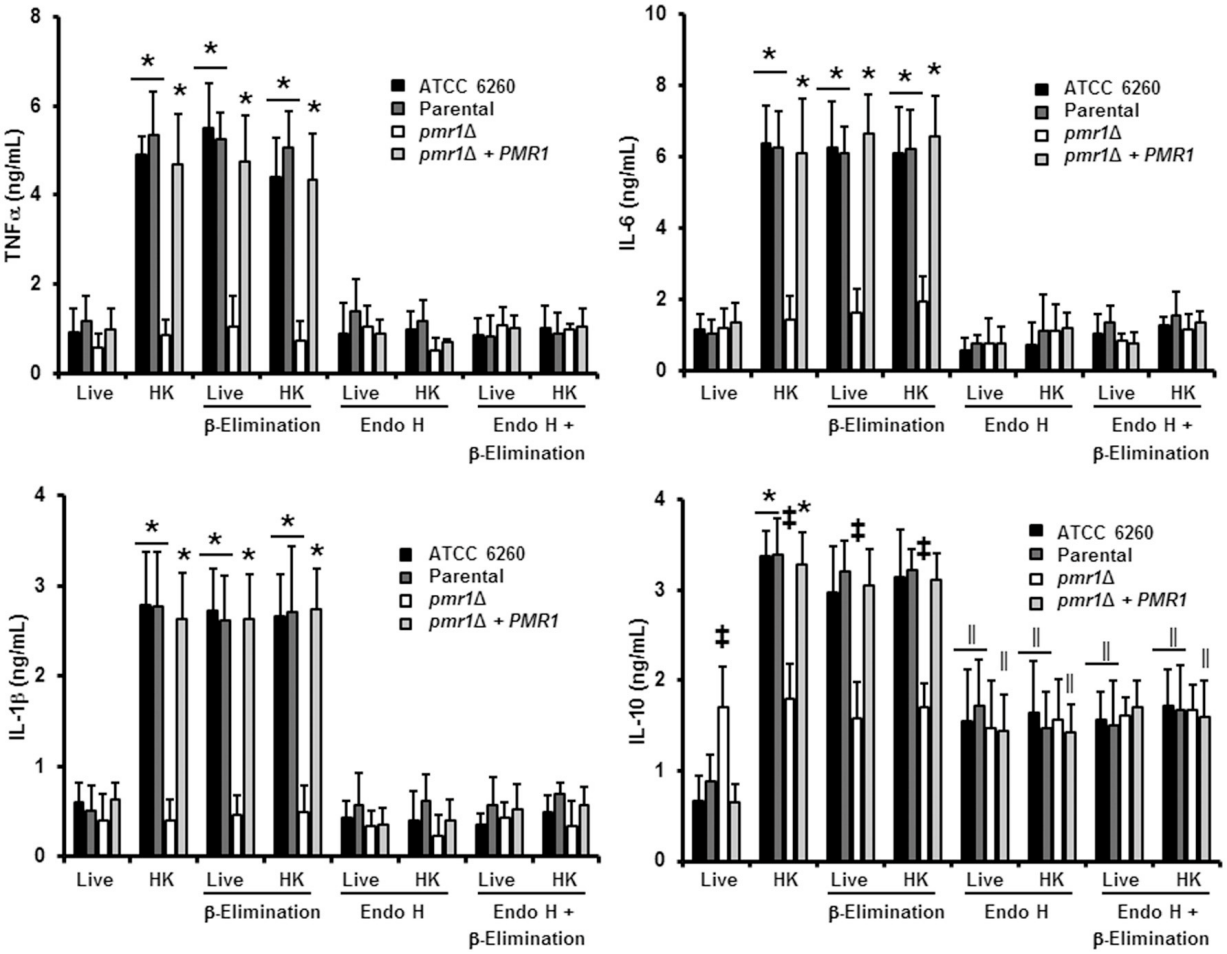
**The loss of proper cell wall mannosylation affects the ability of ***C. guilliermondii*** to stimulate cytokine production by human PBMCs**. Fungal cells were co-incubated with human PBMCs, the supernatant saved and used to quantify pro- and anti-inflammatory cytokines. Results (means ± SD) were obtained using samples from six donors, each assayed in duplicate wells. The strains used are: ATCC 6260, KU141 (Parental), HMY134 (*pmr1*Δ) and HMY138 (*pmr1*Δ + *PMR1*) ^*^P < 0.05, when compared with live cells; ^‡^*P* < 0.05, when compared with cells subjected to the same treatment; ^||^*P* < 0.05, when compared with untreated yeast cells.

We next assessed the relevance of protein mannosylation during the host-fungus interaction, analyzing the ability of *C. guilliermondii* to stimulate cytokine production by human PBMCs. Live *C. guilliermondii* cells from control strains stimulated low and similar TNFα, IL-1β, IL-6, and IL-10 levels (Figure 6); however, when heat inactivated, both strains stimulated significantly higher levels of these cytokines (Figure 6). Live *pmr1*Δ null mutant induced similar levels of TNFα, IL-1β, and IL-6, when compared to live control cells, but these levels showed no increment upon heat killing (Figure 6). Importantly, the re-integrant control strain stimulated cytokine production at similar levels to those generated with the other control strains (Figure 6). For IL-10 production, the *pmr1*Δ null mutant stimulated similar and intermediate levels of this cytokine in both live and HK forms; which were significantly different when compared with those induced with the control cells: higher in the case of live cells and lower when HK cells were used (Figure 6). In accord with these observations, cells with mannoses shaved by endo H and β-elimination, with low levels of mannan on the cell wall (Figure 3) produced cytokine levels similar to that of the *pmr1*Δ null mutant, stressing the relevance of both *N*-linked and *O*-linked mannans for maximal stimulation of TNFα, IL-6, and IL-1β (Figure 6). Furthermore, these results also confirmed that loss of both types of mannans disrupts the proper IL-10 stimulation (Figure 6). The null mutant cells treated with endo H and β-eliminated displayed the same ability to stimulate cytokine production than the untreated, null mutant cells (Figure 6). Therefore, *N*-linked and *O*-linked mannosylation in *C. guilliermondii* are directly linked to the stimulation of cytokines by human PBMCs.

### The *O*-linked mannans negatively affect the recognition of *C. guilliermondii* by human PBMCs

Since the double treated cells with endo H and β-elimination displayed a similar ability to stimulate cytokine production as the *pmr1*Δ null mutant strain, and β-elimination has been recently used to explore the role of *O*-linked mannans in the *C. parapsilosis*-host interaction (Estrada-Mata et al., 2015; Pérez-García et al., 2016), we next examined the importance of fungal *O*-linked mannans during the interaction between human PBMCs and *C. guilliermondii*. Upon β-elimination, both parental (KU141) and re-integrant control strains tended to lose about 35% of cell wall mannan. This modification was accompanied with a significant increment in the content of β-glucan (Table 1). Conversely, the *pmr1*Δ null mutant only showed a higher content in β-glucan levels (Table 1). Live ATCC 6260, parental (KU141), and re-integrant control strains stimulated higher TNF-α, IL-6, IL-1β, and IL-10 levels than those challenged with untreated cells, and these cytokine levels were as high as those measured with untreated-HK cells (Figure 6). β-Elimination did not affect the cytokine levels induced by HK control or mutant cells, nor the levels measured after challenge with the *pmr1*Δ null mutant (Figure 6). Therefore, these data suggest that *O*-linked mannans play a minor role in the stimulation of the analyzed cytokines. This indicates that these oligosaccharides mask other *C. guilliermondii* cell wall components responsible for the stimulation of a strong cytokine production.

**Figure 7:**
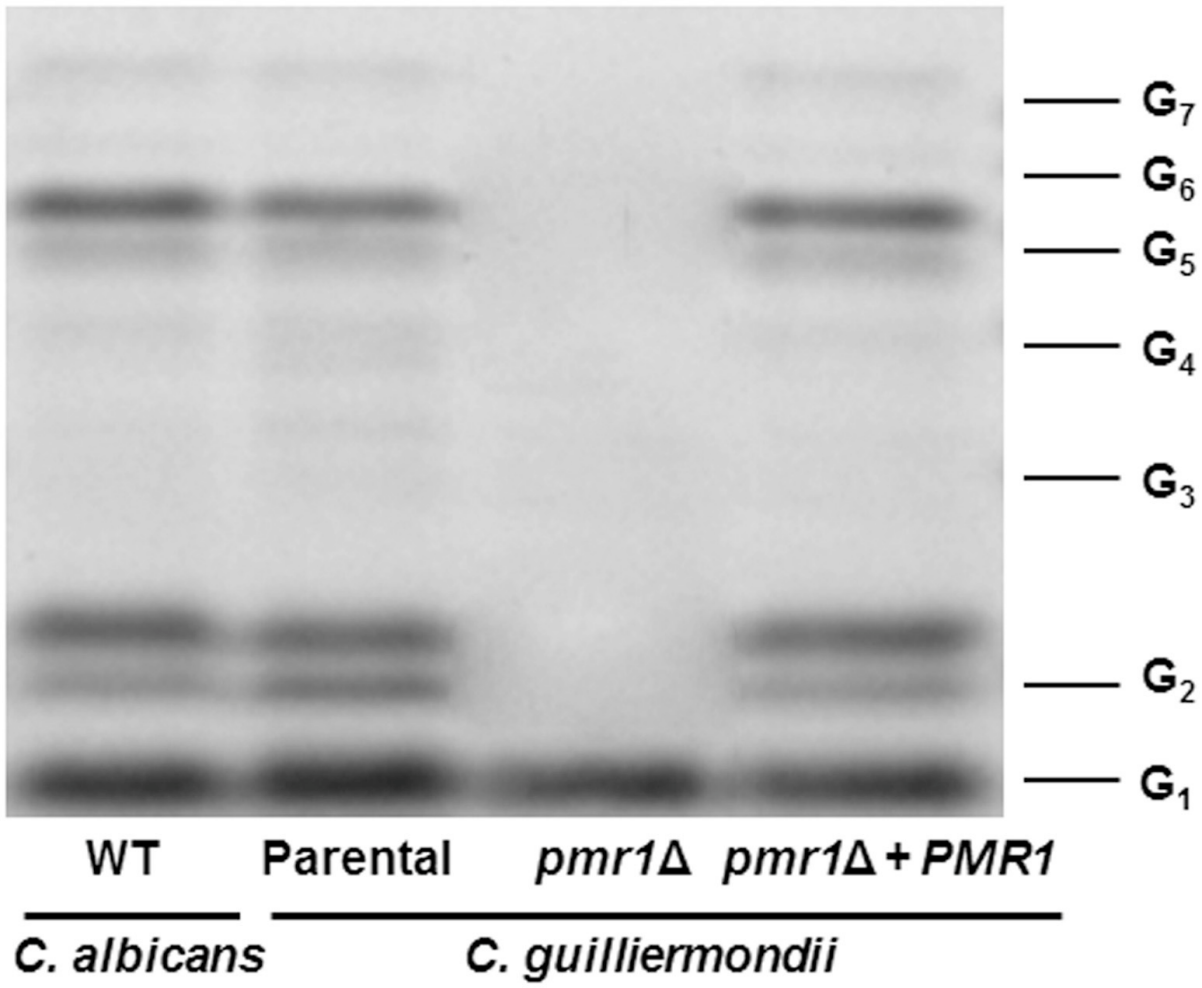
**Fluorophore-assisted carbohydrate electrophoresis of ***O***-linked mannans**. Upon β-elimination, *O*-linked mannans were derivatized with 8-aminonaphthalene-1,3,6-trisulfonic acid and sodium cyanoborohydride and separated in a 35% (w/v) polyacrylamide gel under non-denaturing conditions. The image is representative of three independent experiments. The strains used are NGY152 (WT) KU141 (Parental), HMY134 (*pmr1*Δ), and HMY138 (*pmr1*Δ + *PMR1*). The molecular marker used was a ladder of maltooligosaccharides from one (G1) to seven (G7) glucose units.

Analysis of the *O*-linked mannans collected after β-elimination by fluorophore-assisted carbohydrate electrophoresis indicated the presence of glycans containing from one to seven mannose units in samples from the parental (KU141) and re-integrant control strains (Figure 7). This electrophoretic profile was similar to that observed for *O*-linked mannans isolated from *C. albicans* (Figure 7). The sample from the *pmr1*Δ null mutant showed only one single band migrating at the same level as a single glucose residue (Figure 7).

### The *N*-linked mannans are key components to stimulate cytokine production by human PBMCs

Following a similar strategy to study the contribution of *O*-linked mannans during *C. guilliermondii*-PBMC interaction, we treated the fungal cells with endo H to trim *N*-liked mannans from the cell wall (Figure 3) and used these cells to analyze the cell wall composition and to stimulate cytokine production by human PBMCs. The cell wall of the parental and re-integrant control strains showed a significant reduction in the mannan content and increment in β-glucan levels after trimming of *N*-linked mannans. However, the *pmr1*Δ null mutant cell wall barely showed mannan levels upon deglycosylation with endo H (Table 1). Results in Figure 6 clearly indicate that the removal of *N*-linked mannan from the cell wall of live *C. guilliermondii* ATCC 6260 cells did not change levels of TNF-α, IL-6, and IL-1 β compared to PBMC exposure to untreated yeast cells. A similar result occurred with the other control strains. In the case of HK cells, the three control strains, ATCC 6260, parental (KU141) and re-integrant, stimulated lower levels of pro-inflammatory cytokines, comparable to those stimulated with live cells (Figure 6). The treatment with endo H did not affect the ability of the *pmr1*Δ null mutant to stimulate cytokine production (Figure 6). The lack of *N*-linked mannans did not affect the levels of IL-10 induced by either the live or HK *pmr1*Δ null mutant; but live and HK control strains stimulated similar levels of IL-10 as the null mutant, which were significantly different from those stimulated by untreated cells (Figure 6). Taken together, these data reveal that *C. guilliermondii N*-linked mannans are key cell wall components for inducing the production of cytokines by human PBMCs.

### Dectin-1 is required for cytokine production stimulated by *C. guilliermondii*

**Figure 8:**
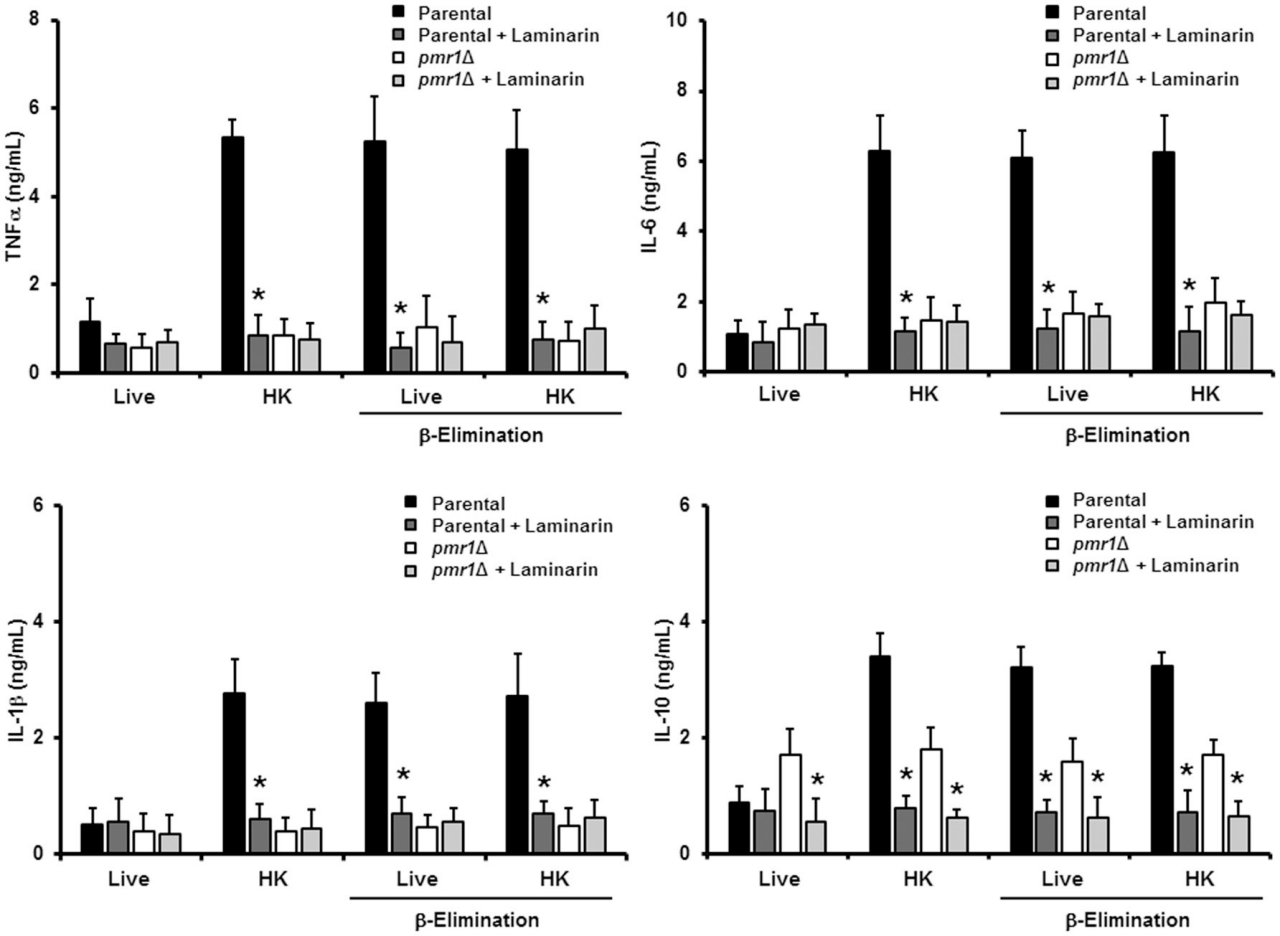
**Blocking of dectin-1 affects human PBMC cytokine induction in response to ***C. guilliermondii*****. Human PBMCs were pre-incubated with laminarin for 1 h at 37°C prior to co-culture with yeast cells. After 24 h incubation at 37°C, the supernatants were saved and used to quantify cytokine levels. Results (means ± SD) were obtained using samples from six donors, each assayed in duplicate wells. The strains used are: KU141 (Parental) and HMY134 (*pmr1*Δ). ^*^P < 0.05, when compared to the same yeast cell type without treatment.

Since mannan sensing is required for a strong cytokine production in response to *C. guilliermondii*, we next analyzed the contribution of β1,3-glucan recognition during the *C. guilliermondii*-human PBMCs interaction. Upon pre-incubation of the human innate immune cells with laminarin, the production of TNFα, IL-6, IL-1β, and IL-10 stimulated by live parental (KU141) cells did not significantly change, when compared with the system in absence of laminarin (Figure 8). However, laminarin blocked the cytokine production when cells were challenged with HK parental, live β-eliminated parental or HK β-eliminated parental cells (Figure 8). Similar results were observed when the ATCC 6260 or the re-integrant control strains were used (Figure 3S). No changes in the levels of TNFα, IL-6, and IL-1β were observed in cells pre-treated with laminarin and exposed to the *pmr1*Δ null mutant; however, IL-10 levels were significantly reduced when cells were stimulated with either live or HK mutant cells with or without β-elimination. Altogether, these results clearly indicate that recognition of β1,3-glucan via dectin-1 strongly influences the cytokine production in response to *C. guilliermondii*.

### Interaction of *C. guilliermondii* with murine macrophages

**Figure 9:**
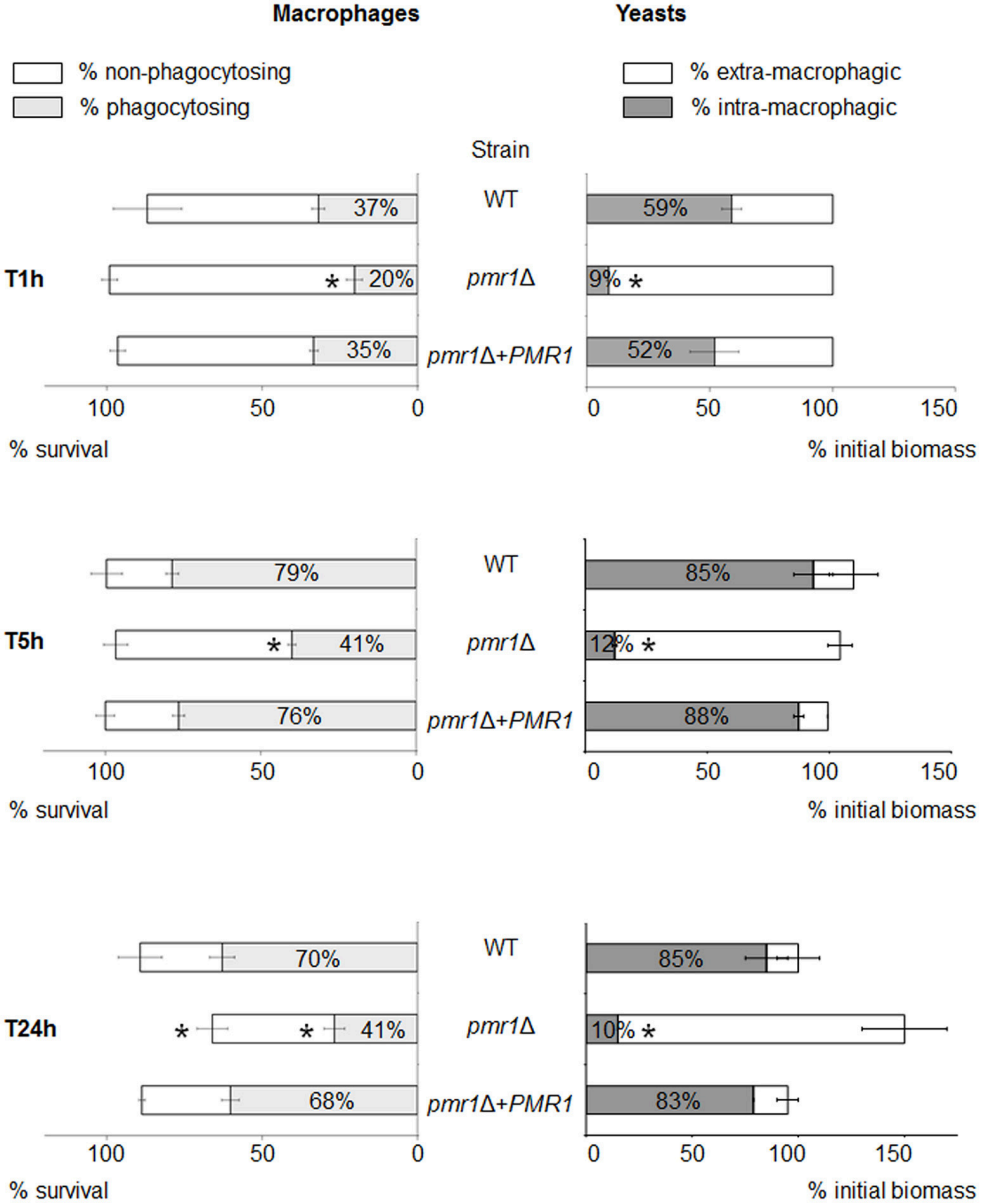
**Multi-parametric analysis of murine macrophage interaction with ***C. guilliermondii*** cells over 24-h time course experiments**. Macrophages were infected with the parental (KU141), the *pmr1*Δ null mutant or the re-integrant (*pmr1*Δ + *PMR1*) strain as described in Materials and Methods. The diagram shows the flow cytometer analysis of the infected macrophages on the left part. The macrophage survival is indicated by the horizontal bar. The white part of the bar represents the percentage of non-phagocytosing macrophages; the gray part represents the percentage of macrophages engaged in phagocytosis. The right part of the diagram shows the fluorometry analysis of the fungal population. The horizontal bar represents the multiplication of the yeast cells in the presence of macrophages, expressed as a percentage of the initial biomass at T1h. The white part of the bar shows the percentage of extra-macrophagic yeasts, the gray part shows the percentage of yeast cells internalized within the macrophages. Each condition was performed in quintuplet (flow cytometry experiments) or in triplicate (fluorescence quenching experiments) per experiment. Each bar is the average of three independent experiments ± standard error. Unpaired *T*-test was used to establish statistical significance. ^*^P < 0.005.

We next assessed the cellular interactions of the *C. guilliermondii* strains with murine macrophages. For this purpose, we used a recently developed *in vitro* model, allowing monitoring of multiple parameters for both cell types over time (Dementhon et al., 2012). In the case of the control strains (parental and re-integrant cells), about 90% of the macrophages survived after 24 h of infection (Figure 9, left panels, and Figure 4S). When infected with the *pmr1*Δ null mutant, macrophages survival was lower (about 70%). The proportion of macrophages engaged in phagocytosis was lower when infected with the *pmr1*Δ null mutant compared to the control strains (20% vs. ~40% at T 1 h, and 40% vs. 70% respectively at T 24 h, Figure 9, left panels, and Figure 4S). Concerning the fungal biomass internalization by the macrophages, Figure 9 clearly shows a lower uptake of the *pmr1*Δ null mutant cells when compared to the control cells. At T 24 h, about 80% of the controls cells were internalized; whereas most of the *pmr1*Δ null mutant cells were outside macrophages. Taken together, our data reveal that loss of *PMR1* significantly affects the interaction of *C. guilliermondii* cells with murine macrophages.

### *C. guilliermondii PMR1* is required for virulence in both *Galleria mellonella* and the mouse model of systemic candidiasis

**Figure 10:**
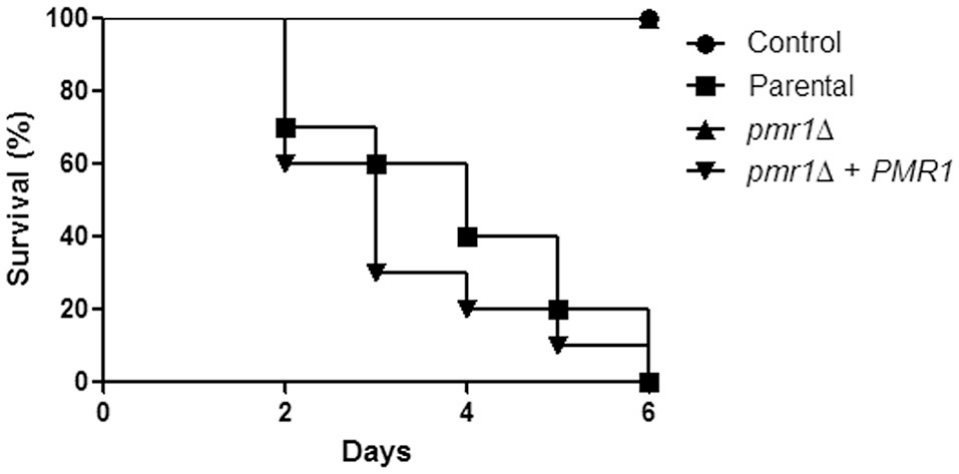
**The ***C. guilliermondii pmr1***Δ null mutant displays virulence attenuation in the ***G. mellonella*** model**. Inocula containing 2 × 10^7^ yeast cells were injected directly into the hemocoel of *G. mellonella* larvae and survival was monitored daily. Experiments were performed three times, with a total of 30 larvae per group (10 larvae for each experiment). Control, PBS-injected group. The strains used are: KU141 (Parental), HMY134 (*pmr1*Δ), and HMY138 (*pmr1*Δ + *PMR1*). No significant differences were observed between the mortality associated to parental and *pmr1*Δ + *PMR1* strains (*P* = 0.32). The *pmr1*Δ null mutant strain showed a significant difference in the ability to kill larvae when compared to the parental or re-integrant control strains (*P* < 0.05 in both cases).

**Figure 11:**
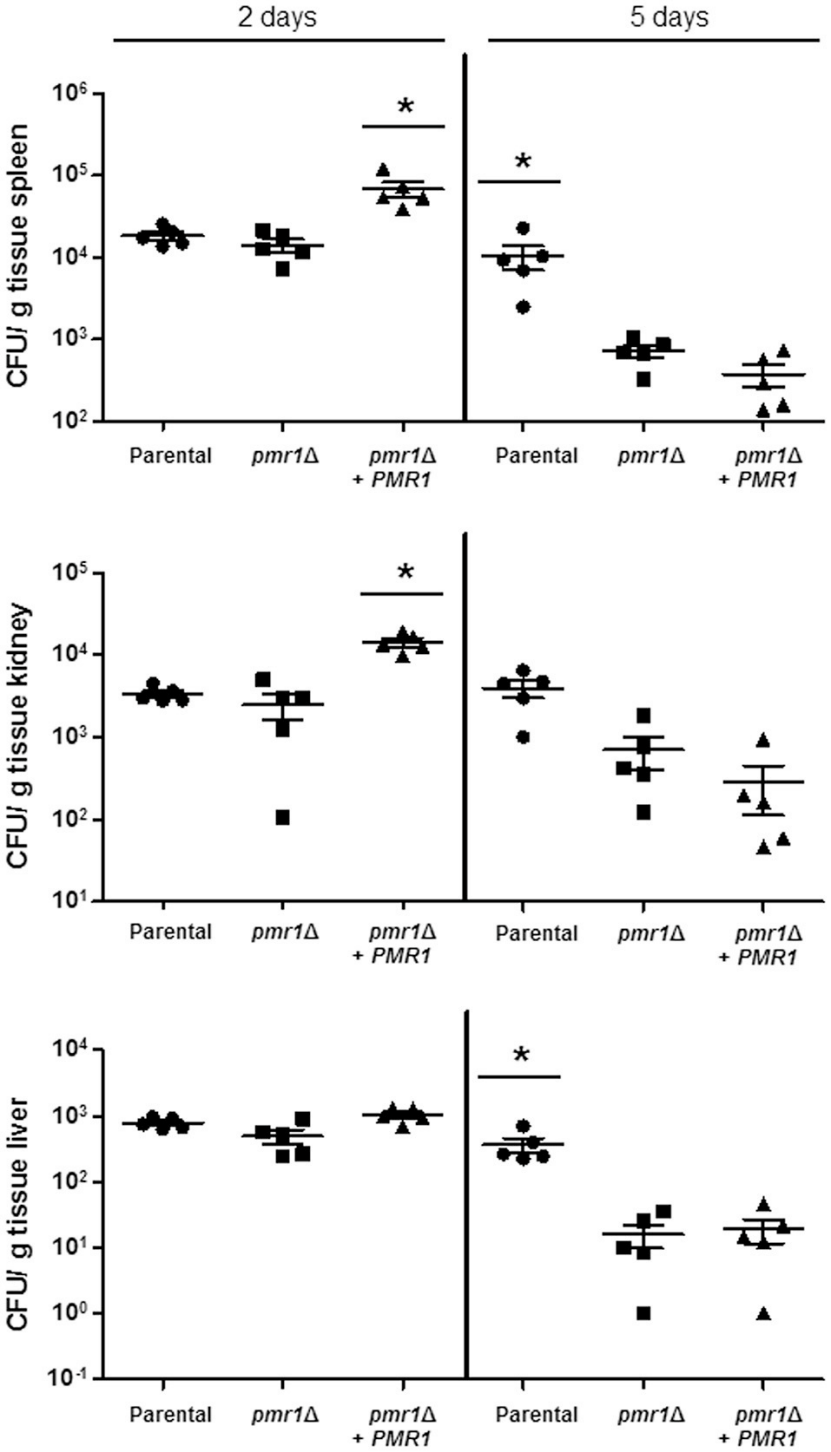
**The ***C. guilliermondii pmr1***Δ null mutant has decreased virulence in the mouse model of systemic candidiasis**. Wild-type BALB/c mice were infected i.v. with 2 × 10^7^ cells of either *C. guilliermondii* KU141 (Parental), HMY134 (*pmr1*Δ), and HMY138 (*pmr1*Δ + *PMR1*), and the fungal burdens in the spleen, kidneys and liver were determined at 2- or 5-days post-infection. Data are expressed as colony forming units (CFU)/g tissue (mean ± SEM). Results are pooled data from 2 separate experiments with 5 mice per group. ^*^P < 0.05.

Finally, to establish the role of protein mannosylation in the *C. guilliermondii* virulence, we assessed the capacity of this yeast to cause disease in *G. mellonella* larva and mice. The parental (KU141) and the re-integrant control strain killed all of the *G. mellonella* larvae by the sixth day post-inoculation, whereas all larvae infected with the *pmr1*Δ null mutant remained alive during the observation period, which was prolonged to 12-day post-inoculation (Figure 10). Furthermore, fungal burden was significantly reduced in animals inoculated with the *pmr1*Δ null mutant (1.5 × 10^8^ ± 0.4 cfu/g, 5.8 × 10^8^ ± 0.9 cfu/g, and 5.3 × 10^8^ ± 1.1cfu/g, for the *pmr1*Δ null mutant, parental (KU141), and the re-integrant control strain, respectively; *P* < 0.05 when compared the burden associated to the null mutant with the control strains). We next used a non-lethal murine experimental model of disseminated candidiasis as previously reported (Ifrim et al., 2014; Pérez-García et al., 2016). At 2 days post-infection, BALB/c mice infected with the *pmr1*Δ null mutant had similar fungal burdens in the spleen, kidneys and liver, as that measured in organs from mice infected with the parental strain (Figure 11). Interestingly, the fungal burden in the spleen and kidneys, but not in liver, was significantly higher at this time interval in animals infected with the re-integrant control strain (Figure 11). At 5-day post-infection, *pmr1*Δ challenged-mice showed a significant reduction in the fungal burdens in the spleen, kidneys and liver, compared to animals infected with the parental yeast cells. Again, it was interesting that, the group of animals infected with the re-integrant control strain at 5 days-post infection displayed fungal burdens that were similar to that measured in mice infected with the null mutant strain (Figure 11). Therefore, these results confirm that the loss of *PMR1* significantly affects the virulence of *C. guilliermondii in vivo*.

## Discussion

A thorough search in the literature and available databases indicates that there are a limited number of disrupted genes in *C. guilliermondii*, and most of them have been used as markers to develop gene manipulation techniques (Millerioux et al., 2011a,b; Foureau et al., 2013a,c; Papon et al., 2013). Our work now presents the first *C. guilliermondii* null mutant with defects in protein mannosylation, in the cell wall and interaction with the host. The herein reported bioinformatics analysis, the heterologous complementation in *C. albicans* and the inability of the *C. guilliermondii pmr1*Δ null mutant to grow in presence of EGTA strongly suggest that the ORF we have analyzed is the functional ortholog of *C. albicans PMR1*. It is noteworthy that *C. guilliermondii PMR1* was controlled by its native promoter when introduced into the *C. albicans pmr1*Δ null mutant, indicating that the transcriptional machinery and the *cis* elements within this promoter are similar between these two organisms, despite their belonging to different subclades of the *Candida* clade (Butler et al., 2009).

Using epifluorescence microscopy, we demonstrated that *C. guilliermondii* Pmr1 tagging with the yellow fluorescent protein did not affect the enzyme activity, as the phenotypes associated with loss of *PMR1* were restored in the re-integrant control strain. Moreover, the fluorescent chimeric protein displayed a compartmentalized intracellular pattern, which most likely represents its distribution within the Golgi complex, as previously reported in *Saccharomyces cerevisiae* Pmr1 (Huh et al., 2003). This strategy did not affect protein localization in *S. cerevisiae*, but its activity was not studied. As previously mentioned, the parental strain used here is in fact a *ku70*Δ null mutant, and thus it is feasible to conceive that any of the analyzed phenotypes can be associated to loss of *KU70* instead of disruption of *PMR1*. However, this is an unlikely scenario, since the parental strain displayed similar phenotypic traits to the ATCC 6260 reference strain, and we observed restoration of the phenotype in the re-integrant control strain.

The *C. guilliermondii pmr1*Δ null mutant has characteristic phenotypes associated with disruption of protein mannosylation pathways: increased duplication rates, abnormal cell morphology, decreased mannan content, and defects in the cell wall composition, organization, porosity and fitness (Bates et al., 2005, 2006, 2013; Munro et al., 2005; Mora-Montes et al., 2007, 2010; Hall et al., 2013; Pérez-García et al., 2016). Interestingly, we observed pseudohyphae formation in the *C. guilliermondii pmr1*Δ null mutant, which has not been shown for the *C. albicans* or *S. cerevisiae pmr1*Δ null mutants (Antebi and Fink, 1992; Bates et al., 2005). The *S. cerevisiae pmr1*Δ null mutant has an increase in intracellular calcium (Yu et al., 2012), and high concentrations of this ion are required to maintain tip enlargement of *C. albicans* hyphae (Brand et al., 2007). So, it is possible that reorganization of calcium levels within *C. guilliermondii pmr1*Δ null mutant affected the radial growth of cells and a polarized growth was established instead. Alternatively, since cells tended to form aggregates, it is possible that the polarized growth is established in regions where the cell surface is not in direct contact with other cells. Nevertheless, this observation requires further analysis to properly understand this phenotypic change.

The role of protein mannosylation in the ability of *Candida* spp. to form biofilms has been poorly explored. Here we found that disruption of this biosynthetic machinery negatively affected the ability of *C. guilliermondii* to form biofilms, and these data are in agreement with the adverse effect of tunicamycin (an inhibitor of the early steps during *N*-linked mannan elaboration) on *C. albicans* biofilms (Pierce et al., 2009). Since cell wall protein diversity is likely to be affected in the *C. guilliermondii pmr1*Δ null mutant, it remains to be addressed whether changes in the sugar moieties or the proteins are behind this observation.

*C. albicans* phosphomannosylation occurs in *O*-linked mannans as well as in the *N*-linked mannan core and its outer chain, and the loss of Pmr1 significantly affected the fungus' ability to bind Alcian blue (Bates et al., 2005, 2006; Mora-Montes et al., 2007). The *C. guilliermondii pmr1*Δ mutant was unable to bind Alcian blue; thus, it is reasonable to suggest that phosphomannan is absent from the *N*-linked mannan core, as ability of the *C. albicans pmr1*Δ null mutant to bind this dye was associated with this oligosaccharide moiety (Bates et al., 2005). The reduced susceptibility to fluconazole associated with the loss of Pmr1 was unexpected, as *S. cerevisiae pmr1*Δ has been shown to display hypersensitivity to this antifungal drug (Kapitzky et al., 2010). Since we do not observe a similar result with nystatin, it is unlikely that general cell wall rearrangements are responsible for the resistance to fluconazole. Instead, the results suggest that specific molecular components involved in the resistance to fluconazole are dysregulated.

Cell wall mannans and β1,3-glucan play a pivotal role during the *C. albicans*-innate immune system interaction (Martínez-Álvarez et al., 2014), and truncation of *C. albicans* mannans reduces cytokine production by human PBMCs (Netea et al., 2006; Mora-Montes et al., 2007, 2010). Similarly, HK *C. guilliermondii pmr1*Δ null mutant or endo-H-treated and β-eliminated WT cells induced lower levels of TNFα, IL-6, and IL-1β production by PBMCs compared to untreated WT cells, indicating that mannans indeed also have a significant immunostimulatory effect. In contrast, truncated cell wall mannans positively affected the stimulation of IL-10, and the increased IL-10 production associated with live *pmr1*Δ null mutant cells was blocked with the addition of laminarin, indicating this was a dectin-1 dependent process. However, the IL-10 levels stimulated by the HK null mutant cells were lower than those associated with the control strains. A similar observation was found in cells lacking proper *N*-linked mannan elaboration in *C. albicans* (Mora-Montes et al., 2010), and together, these data indicate that, as in *C. albicans*, stimulation of IL-10 production in *C. guilliermondii* can be performed via dectin-1, but both, mannan and β1,3-glucan recognition are required for a strong IL-10 production (Netea et al., 2006; Gow et al., 2007; Reid et al., 2009; Mora-Montes et al., 2010). Treatment of *C. guilliermondii* with endo-H indicated that *N*-linked mannans play a similar role in cytokine production by human PBMCs as that reported in *C. albicans*, in which exposure of β1,3-glucan by heat killing exposed key cell wall components that induced high levels of pro-inflammatory cytokines (Netea et al., 2006; Mora-Montes et al., 2007, 2010). Therefore, it is likely that wall components are masking the β1,3-glucan layer in *C. guilliermondii*, which effectively inhibit the triggering of cytokine production via dectin-1, as reported in *C. albicans* and *C. parapsilosis* (Wheeler and Fink, 2006; Gow et al., 2007; Pérez-García et al., 2016).

It is interesting that, despite the *pmr1*Δ null mutant has more β1,3-glucan exposed at the cell wall, this did not lead to a higher stimulation of cytokines. Similar observations have been reported for *C. albicans* and *C. parapsilosis* null mutants with defects in mannan elaboration and higher levels of β1,3-glucan at the cell wall (Netea et al., 2006; Mora-Montes et al., 2007, 2010; Pérez-García et al., 2016). These data suggest that, although recognition of β1,3-glucan via dectin-1 is important for the stimulation of cytokine production in human PBMCs, this ligand-receptor interaction should be part of a co-stimulation network where other receptors, along with dectin-1, are triggering the production of significant levels of cytokines. Indeed, it has been reported that dectin-1 collaborates with TLR-2, TLR-4, TLR-5, TLR-7, and TLR-9 in a synergistic way to induce cytokine synthesis (Reid et al., 2009). Furthermore, it was recently reported that dectin-1 is important during the sensing of *C. albicans*, but dispensable, as some *C. albicans* strains with high content of cell wall chitin are not recognized by this receptor (Marakalala et al., 2013). Therefore, it is likely this co-stimulation network, involving dectin-1, also occurs during the immune sensing of *C. guilliermondii*. The *C. albicans O*-linked mannans account for a minor component of the cell wall (Munro et al., 2005), and are dispensable for cytokine stimulation by human PBMCs (Netea et al., 2006). Here, we also observed that *C. guilliermondii* cells lacking *N*-linked mannans, but displaying *O*-linked mannans in the cell wall (endo-H treated cells), were unable to stimulate a robust cytokine response, despite β1,3-glucan being exposed at the cell surface. However, the chemical removal of *O*-linked mannans (β-eliminated cells) from *C. guilliermondii* control cells resulted in the induction of pro- and anti-inflammatory cytokines in a manner similar to that observed with *C. guilliermondii* HK cells. Our fluorophore-assisted carbohydrate electrophoresis of *C. guilliermondii O*-linked mannans showed that the abundance of glycans with different length is similar to that found in *C. albicans O*-linked mannans, with structures composed from one to seven residues. This technique has the sensitivity to discriminate between oligosaccharides with the same size, but composed of sugars bound with different glyosidic linkages (Jackson, 1990). Thus, the intense bands, with lower migration than the molecular marker are likely to be *O*-linked mannans bearing a phosphomannan residue, which has been described in *C. albicans* (Mora-Montes et al., 2007). These data also suggest that the *O*-linked mannans from *C. guilliermondii* should contain α1,2-mannose units, as described in *C. albicans* (Munro et al., 2005). The presence of *O*-linked mannans with one mannose residue in the *pmr1*Δ null mutant was expected, as this is added in the endoplasmic reticulum (Munro et al., 2005). Since cell wall *O*-linked mannans are more abundant (accounting for about one third of the total mannan content) in *C. guilliermondii* than in *C. albicans*, it is possible to suggest that, like *N*-linked mannans, these oligosaccharides are masking inner cell wall components of *C. guilliermondii* from recognition by immune receptors. Along this line, a new and differential role for *O*-linked mannans has been recently established during *C. parapsilosis*-human PBMCs interaction (Estrada-Mata et al., 2015; Pérez-García et al., 2016). It is noteworthy that β-elimination or endo-H treatment, besides reducing mannan content, positively affected the glucan levels, but not the chitin content. The cell wall integrity pathway compensates defects in the cell wall composition, after interaction with cell wall perturbing agents or disruption of genes involved in wall biosynthesis (Dichtl et al., 2016). In *C. albicans*, when mannan assembly is disrupted, the activation of this pathway leads to increased levels of both chitin and β-glucan (Bates et al., 2006, 2013; Mora-Montes et al., 2007, 2009, 2010). In the experimental conditions of deglycosylation, cells are not suspended in culturing medium, and only rely on the internal storages of nutrients and energy; therefore, it is possible to suggest that *C. guilliermondii* is incapable to perform a full wall adaptation to the deglycosylation process, implying that the energy cost of glucan synthesis is lower than chitin elaboration. Alternatively, it is also possible that the components of the cell wall integrity pathway in *C. guilliermondii* are not the same, or perform different functions, and the removal of mannan does not lead to an increment in chitin levels. Nevertheless, further studies are required to elucidate the components of this signaling pathway in *C. guilliermondii*.

The analysis of the interaction of *C. guilliermondii* with macrophages showed that the *pmr1*Δ null mutant was lesser internalized than control strains, suggesting a key role for mannans during the uptake by these phagocytic cells. In line, a similar observation was reported in *C. albicans*, where lack of cell wall mannans negatively affected uptake and phagocytosis (McKenzie et al., 2010). Interestingly, we observed more death macrophages after 24 h interaction with the null mutant cells when compared to the control strains. This may be attributable to the cell morphology of the mutant, as cell aggregates are likely to damage macrophage membranes than single cells. Alternatively, it is possible to suggest that the cell wall rearrangements could expose molecules with toxic effects on the phagocytic cells.

The *C. guilliermondii pmr1*Δ null mutant had significantly decreased virulence in both *G. mellonella* and murine models of systemic candidiasis, similar to that reported for *C. albicans* (Bates et al., 2005). However, in the mouse model the re-integrant strain failed to colonize and kill as the parental control cells, recuperating similar CFU from organs to those obtained from animals infected with the null mutant strain. The backbone vector used to generate the *pmr1*Δ + *PMR1* re-integrant strain did not integrate into the *C. guilliermondii* genome, as it contains an autonomously replicating sequence (Foureau et al., 2013a), so it is tempting to speculate that cells growing in a rich nutrient condition such as that which is present in the mouse milieu tend to lose the plasmid containing *PMR1*. Indeed, when this strain was grown in rich medium, like YPD or Sabouraud, the cells tended to show phenotypic traits of the null mutant strain (data not shown). Therefore, as previously demonstrated (Defosse et al., 2014), growth on minimal medium is required to force this strain to keep the plasmid that restores *PMR1* mutation. Nevertheless, our results clearly show virulence attenuation in the mouse model upon *PMR1* disruption.

In conclusion, we report that *PMR1* regulates protein mannosylation in *C. guilliermondii* and the affected mannosylation processes are relevant during interaction with the host. Loss of *PMR1* in *C. guilliermondii* leads to morphological alterations, defects in the ability to form biofilms, and aberrant wall composition and organization. Furthermore, *N*-linked and *O*-linked mannans have differential roles during cytokine stimulation when *C. guilliermondii* cells interact with innate immune cells. Finally, our results demonstrate that *C. guilliermondii*, although is closely related to *C. albicans*, has subtle but key differences in its biology, which makes it an important model to study at both molecular and immunological levels.

## Experimental procedures

### Strains and growth conditions

Organisms generated and used in this study are listed in Table 2. Unless otherwise indicated, cells were maintained and propagated at 30°C in SC medium [0.76% (w/v) yeast nitrogen base without amino acids, 2% (w/v) glucose] supplemented with 50 μg/mL uridine when required. To prepare cells for cytokine assays and cell wall analysis, the strains were grown at 30°C in 500 mL flasks containing 100 mL of fresh medium with shaking at 200 rpm until reaching mid-log phase of growth. Cells were HK by incubating at 56°C for 60 min. Loss of cell viability was confirmed by an absence of growth on SC plates after 48 h incubation at 28°C. For β-elimination assays, cell samples were treated as previously described (Díaz-Jiménez et al., 2012). Briefly, cells were suspended in 10 ml of NaOH 0.1 N and incubated at room temperature during 18 h with gently shaking. The reaction was stopped by neutralizing with HCl 0.1 N, and cells were pelleted at 2000 × g, for 5 min. The supernatant was saved for *O*-linked mannans analysis (see below). The *N*-linked mannan removal was achieved by incubating at 37°C with 25 U endo H (New England Biolabs) as reported (Mora-Montes et al., 2012). In both cases, cells were washed twice with sterile phosphate buffered saline (PBS) and viability was demonstrated by the lack of significant differences in the number of cfu/mL before and after the treatment (loss of cell viability upon β-elimination and endo H treatment was 3.6 ± 1.9% and 5.6 ± 3.7%, respectively). Cell aggregates were dispersed by the addition of 2 units/mL chitinase (Sigma) to medium (Bates et al., 2006; Pérez-García et al., 2016).

**Table 2:** **Strains used in this work**.

**Strain**	**Organism**	**Origin**	**Genotype**	**References**
ATCC 6260	*C. guilliermondii*	ATCC	Wild type	ATCC
KU141	*C. guilliermondii*	NP566U	*ura5, ku70*::*REP-URA5-REP*	Foureau et al., 2013b
KU141F1	*C. guilliermondii*	Derived from KU141	*ura5, ku70*::*REP*	Foureau et al., 2013b
HMY134	*C. guilliermondii*	Derived from KU141F1	*ura5, ku70*::*REP, pmr1*Δ::REP-*URA5*-REP	This work
HMY136	*C. guilliermondii*	Derived from HMY134	*ura5, ku70*::*REP, pmr1*Δ::REP	This work
HMY138	*C. guilliermondii*	Derived from HMY136	*ura5, ku70*::*REP, pmr1*Δ::REP + pGURA5-*PMR1*-YFP	This work
HMY188	*C. guilliermondii*	Derived from HMY136	*ura5, ku70*::*REP, pmr1*Δ::REP + pGURA5-YFP-*PMR1*	This work
NGY152	*C. albicans*	Derived from CAI-4	*ura3*Δ-*iro1*Δ::*imm434*/ *ura3*Δ-*iro1*Δ::*imm434*; *RPSI*/*rps1*Δ::Clp10	Brand et al., 2004
NGY98	*C. albicans*	Derived from NGY97	*ura3*Δ-*iro1*Δ::*imm434*/ *ura3*Δ-*iro1*Δ::*imm434*; *pmr1*Δ::*hisG*/*pmr1*Δ::*hisG*	Bates et al., 2005
NGY355	*C. albicans*	Derived from NGY98	As NGY98, but *RPSI*/*rps1*Δ::Clp10	Bates et al., 2005
HMY186	*C. albicans*	Derived from NGY98	As NGY205, but *RPSI*/*rps1*Δ::Clp10-Cg*PMR1*	This work

### Construction of the Cg*pmr1*Δ null mutant, and re-integrant control strain

The *C. guilliermondii pmr1*Δ null mutant was constructed as follows. Genomic DNA of *C. guilliermondii* ATCC 6260 reference strain was first extracted and purified using Plant Nucleospin II kit (Macherey-Nagel). This genomic DNA was used to amplify by PCR a 2769 bp fragment (primers PMR11 5′-CTGAGAGGATCCATGACAGAGAACCCGTTCGATGTG-3′ and PMR12 5′-CTGAGAACTAGTTACGCTGTAGCTGACTCCATTCAT-3′) overlapping the Cg*PMR1* coding sequence. PCR reactions were performed with Phusion DNA polymerase (New England Biolabs). This PCR fragment was cloned into pGEM-T easy vector (Promega) to yield pG-Cg*PMR1*. To obtain plasmid pG-*5*′*PMR1-REP-URA5-REP-3*′*PMR1*, pG-Cg*PMR1* was digested with BstBI to delete a 1115 bp central fragment in the *PMR1* coding sequence. The resulting digested plasmid was ligated to the *REP-URA5-REP* fragment released after digestion of the pG-*REP-URA5-REP* plasmid (Foureau et al., 2013b) with ClaI (compatible with BstBI). The *5*′*PMR1-REP-URA5-REP-3*′*PMR1*disruption cassette was released from pGEM-T vector after digestion of the pG-*5*′*PMR1-REP-URA5-REP-3*′*PMR1*with NotI and used to transform the non-homologous end-joining pathway deficient strain KU141F1 (genotype *ura5, ku70*) (Foureau et al., 2013b). Transformation of *C. guilliermondii* cells was performed as described previously (Millerioux et al., 2011a). Ura^+^ transformants were selected on SC medium after 4 days of growth at 35°C. Genomic DNA of a series of randomly selected Ura^+^ transformants was extracted and homologous integration of the disruption cassette at the *PMR1* locus was confirmed by PCR (Figure 2B). A representative null mutant abbreviated *pmr1*Δ was chosen for further phenotype analysis.

The *pmr1*Δ + *PMR1* re-integrant strain was constructed as follows. The codon-optimized sequence of YFP gene was amplified with primers GYCN1 5′-CTGAGAGCTAGCATGTCTAAAGGTGAAGAATTAT-3′ and GYCS2 5′-CTGAGAGTCGACTTTGTACAATTCATCCATACCA-3′ using pYFP-URA3 (Gerami-Nejad et al., 2001)(kindly provided by C.A. Gale, Department of Pediatrics, University of Minnesota, Minneapolis, USA) as template, digested with NheI and SalI and cloned into the corresponding site of the *C. guilliermondii* expression vector pG*-URA5-P*_*ACT1*_*-T*_*TRP1*_ (Defosse et al., 2014) between the *C. guilliermondii ACT*1 promoter (*P*_*ACT1*_) and the *C. guilliermondii* phosphoribosylanthranilate isomerase (*TRP1*) gene terminator (*T*_*TRP1*_) to yield pG-*URA5-YFP*. The 2769 bp PCR-amplified fragment (primers PMR11 and PMR12) overlapping the *PMR1* coding sequence was digested with BamHI and SpeI and cloned into BamHI and NheI (compatible with SpeI) of pG-*URA5-YFP*, in frame of the YFP gene, to yield pG-*URA5-PMR1-YFP*. The 2769 bp PCR-amplified fragment digested with BamHI and SpeI was also cloned into BglII and AvrII (compatible with BamHI and SpeI, respectively) of pG-*URA5-YFP* to yield pG-*URA5-YFP-PMR1*. A representative *pmr1*Δ null mutant was subjected to a FOA counter-selection to select Ura^−^ cells derived from *URA5* marker loss (Millerioux et al., 2011b). Genomic DNA of a series of randomly selected Ura^−^ FOA resistant clones was extracted and the loss of a *REP-URA5* fragment at the *pmr1::REP-URA5-REP* disrupted locus was confirmed by PCR. A representative Ura^−^ null mutant strain (genotype *pmr1::REP*) was finally transformed with circular pG-*URA5-PMR1-YFP* or pG-*URA5-YFP-PMR1*. Ura^+^ transformants were selected on SC medium after 4 days of growth at 35°C. An Olympus BX51 epifluorescence microscope equipped with the Olympus DP71 digital camera and the Cell^*^D imaging software (Soft Imaging System, Olympus) was used for image capture and for merging false-colored images of *C. guilliermondii* colonies expressing the YFP. Details on the combinations of filters sets used for each application are previously described (Courdavault et al., 2011).

### Heterologous complementation in *C. albicans*

The Cg*PMR1* open reading frame, along with 1 Kbp upstream and 600 bp downstream sequences (4307 bp in total) was amplified by PCR using the primer pair 5′- GCGGCCGCGTTCGTGCGACTTTGGA-3′ and 5′- GCGGCCGCGATCATAGGAACATGATGGG-3′ (sequences to generate NotI sites are underlined). The amplicon was cloned into pCR®2.1-TOPO® (Invitrogen), and subcloned into the NotI site of CIp10 (Murad et al., 2000). Upon digestion with StuI this construction was used for transformation of a Ca*pmr1*Δ null mutant (Bates et al., 2005).

### Analysis of biofilm formation

Biofilm formation and its analysis were performed as described (Peeters et al., 2008). Briefly, 100 μL aliquots containing 1 × 10^6^ cells were seeded in flat-bottom polystyrene 96-microtiter plates (NUNC) and allowed to adhere for 4 h at 30°C. Non-adherent cells were removed and wells were rinsed using 100 μL PBS. Then, 100 μL of fresh SC medium were added to each well and the plate was incubated 24 h at 30°C. The medium was discarded, wells were thoroughly rinsed with PBS and cells were fixed with 100 μL of 99% (v/v) methanol. After 15 min incubation at room temperature, the methanol was removed and plates were allowed to air-dry. Aliquots containing 100 μL of 0.02% (w/v) crystal violet were then added to each well and 20 min later plates were rinsed with water. Finally, the reaction was developed by the addition of 150 μL of 33% (v/v) acetic acid and the absorbance was measured at 590 nm. Values generated with the WT strain were considered as 100% and used for data normalization.

### Analysis of cell wall composition

Cells were mechanically broken in a Braun homogenizer during 5 min and under a CO_2_ stream; with cycles of 1 min, with 2-min resting periods on ice. The homogenate was centrifuged and cell walls were recovered, extensively washed with deionized water, cleansed and acid-hydrolyzed as described (Mora-Montes et al., 2007). Acid-hydrolyzed samples were analyzed by HPAEC-PAD in a carbohydrate analyzer system from Dionex, using the separation conditions previously reported (Cheng et al., 2011; Pérez-García et al., 2016).

Cell wall protein content was determined upon wall hydrolysis in alkali (Mora-Montes et al., 2007), using the Bradford protein assay.

Cell wall porosity was assessed by relative porosity to polycations as reported (De Nobel et al., 1990). Briefly, overnight-grown cells were harvested by centrifuging and inoculated into fresh SC broth, incubated for 8 h at 30°C and 200 rpm, and washed twice with PBS. Pellets containing 1 × 10^8^ cells were suspended in either 10 mM Tris-HCl, pH 7.4 (buffer A), buffer A plus 30 μg/mL poly-L-lysine (MW 30–70 kDa, Sigma Cat. No. P-2636) or buffer A plus 30 μg/mL DEAE-dextran (MW 500 kDa, Sigma Cat. No. D-9885), and incubated for 30 min at 30°C with constant shaking at 200 rpm. Cells were pelleted by centrifugation, and the supernatants were collected and measured at an absorbance of 260 nm. The relative cell wall porosity to DEAE-dextran was calculated as described, using the porosity to poly-L-lysine for data normalization (De Nobel et al., 1990).

The content of cell wall phosphomannan was determined by the ability of cells to bind Alcian blue. Cells in exponential growth phase were collected, washed twice with deionized water and adjusted to an OD_600_ of 0.2. Aliquots of 1 mL were pelleted and cells suspended in 1 mL of 30 μg/mL Alcian blue (in 0.02 M HCl) and analyzed as described (Hobson et al., 2004).

### Analysis of cell wall *N*-linked and *O*-linked mannan content

Cells were incubated overnight at 37°C with 25 U endo H (New England Biolabs) to trim cell wall *N*-linked mannans (Mora-Montes et al., 2012). Cells were also β-eliminated as described (Díaz-Jiménez et al., 2012). In both cases, cells were centrifuged, and the supernatant were freeze-dried and used to quantify the sugar content according to a reported the phenol-sulfuric-acid protocol (Dubois et al., 1956). In both cases, mannan release was confirmed by HPAEC-PAD as described (Pérez-García et al., 2016).

### Fluorophore-assisted carbohydrate electrophoresis

Aliquots containing 20 mg *O*-linked mannans were derivatized with 8-aminonaphthalene-1,3,6-trisulfonic acid and sodium cyanoborohydride for at least 16 h at 37°C, as previously described (Jackson, 1990). The material was then dried under vacuum and separated in a 35% (w/v) polyacrylamide gel under non-denaturing conditions, for 6 h at 4°C and 200 V. The gel was inspected under UV light, and the image was captured using the Chemidoc MP system (Bio-Rad). As control, a sample from *C. albicans* NGY152 *O*-linked mannans was analyzed under the same conditions. The molecular marker used was a ladder of maltooligosaccharides from one to seven glucose units (Sigma).

### Fluorochrome staining

Chitin staining was performed as described (Mora-Montes et al., 2011), using 1 mg/mL WGA-FITC (Sigma). The labeling of β1,3-glucan was performed with 5 μg/mL IgG Fc-Dectin-1 chimera (Graham et al., 2006) for 40 min at room temperature, followed by incubation with 1 μg/mL donkey anti Fc IgG-FITC for 40 min at room temperature (Marakalala et al., 2013). The samples were examined by fluorescence microscopy using a Zeiss Axioscope-40 microscope and an Axiocam MRc camera. From the pictures acquired, the fluorescence quantification of 100 cells was achieved using Adobe Photoshop™ CS6 using the formula: [(total of green pixels-background green pixels) × 100]/total pixels. The experiment was performed three independent times, with a total of 300 cells analyzed per strain.

### Susceptibility to cell wall perturbing agents and antifungal drugs

Strains were assessed for susceptibility to wall perturbing agents using the described microdilution method (Bates et al., 2005). Cells were cultivated overnight in SC medium supplemented with 2 units/mL chitinase and then collected and washed with deionized water. The cells were separated using a syringe with a 32-gauge needle, and suspended to an OD_600_ = 1. Then, cells were inoculated into fresh SC medium at an OD_600_ of 0.01 and 95 μL were distributed into the wells of 96-well plates. The cell wall perturbing agents, in a final volume of 5 μL, were added to each well. Control wells received 5 μL of vehicle only and were used to normalize the results. The final OD_600_ for each well was determined after 24 h incubation at 30°C. The maximum concentrations tested for each agent were: 400 μg/mL Calcofluor White (CFW, Sigma), 200 μg/mL Congo Red (Sigma), 300 μg/mL hygromycin B (GoldBio); 50 μg/mL tunicamycin (Sigma) and 0.1% (w/v) SDS (Invitrogen). Growth data were normalized as percentage of those generated with the same strain with no treatment (medium only).

The minimum inhibitory concentration of the antifungal agents was performed according to the CLSI document M27-S4. Samples of 100 μL with 1 × 10^3^ cells from overnight cultures were seeded in round-bottom 96-well plates containing serial dilutions of the tested drugs in RPMI 1640 (Gibco). The initial drug concentration for fluconazole and nystatin were 125 μg/mL and 16 μg/mL, respectively. Plates were incubated at 35°C for 24 h before the minimum inhibitory concentration was determined.

### Ethics statement

The use of human primary cells was approved by the Ethics Committee from Universidad de Guanajuato (permission number 17082011). The primary cells were collected from healthy adult volunteers. The Ethics Committee at the University of Szeged approved the experimentation with mice (XII./00455/2011).

### Isolation and stimulation of human PBMCs with *C. guilliermondii* cells

Human PBMCs were isolated using Histopaque-1077 (Sigma) as reported (Endres et al., 1988). To stimulate cytokine production, 100 μL containing 5 × 10^5^ PBMCs in RPMI 1640 Dutch modification (added with 2 mM glutamine, 0.1 mM pyruvate and 0.05 mg/mL gentamycin; all reagents from Sigma) were plated onto round-bottom 96-well microplates, and 100 μL with 1 × 10^5^ fungal cells freshly harvested or treated were added to each well. The co-cultures were incubated for 24 h at 37°C with 5% (v/v) CO_2_. In some experiments, PBMCs were pre-incubated for 1 h at 37°C with 200 μg/mL laminarin before stimulation with yeast cells. Laminarin used for the pre-incubation experiments was not contaminated with LPS (tested with the *Limulus* amebocyte lysate from Sigma); however, all reactions were performed in presence of 5 μg/mL polymyxin B (Sigma) (Schwartz et al., 1972). The plates were centrifuged for 10 min at 3000 × g at 4°C, and the supernatants were collected and stored at −20°C until used.

### Cytokine quantification

The concentrations of TNFα, IL-6, and IL-10 were quantified by ELISAs (Peprotech), and IL-1β levels were determined using an ELISA kit from R&D Systems.

### Macrophage infection with yeasts

Macrophages from the J774 cell line were infected as described (Dementhon et al., 2012) in cRPMI medium (RPMI-1640 from Sigma without phenol red and supplemented with 10% heat-inactivated FBS, 1 mM sodium pyruvate and 2 g/L sodium bicarbonate) at 37°C under 5% (v/v) CO2. A Multiplicity of Infection (MOI) of 1 macrophage to 1 yeast (1M:1Y) was used for all experiments, except for yeast viability, which we used 10M:1Y. Macrophages (2 × 10^5^ per well) were adhered overnight in 96-well plates and then CFW-labeled yeast cells were added in cRPMI in the presence of 5 μg/mL CFW to allow the continuous labeling of newly replicated yeasts outside the macrophages.

### Fluorimetry and flow cytometry assays

Fluorimetry and flow cytometry assays were conducted as previously described (Dementhon et al., 2012). Fluorimetry was used to determine the multiplication of the fungal biomass and the ratio of ingested fungal cells after 1, 5, and 24 h of infection. Briefly, we measured the total fluorescence of CFW-labeled yeasts per well. The fungal biomass multiplication was determined by comparing the CFW fluorescence at 5 and 24 h with the fluorescence of the initial biomass at 1 h. To determine the uptake of fungal cells by the macrophages, a final concentration of 250 μg/mL trypan blue was used to quench the fluorescence of extracellular CFW-labeled yeasts. The residual fluorescence of internalized CFW-labeled yeasts was measured and expressed as a percentage of the total fluorescence. Flow cytometry was used to measure the macrophage and yeast mortality rates, and the ratio of macrophages engaged in phagocytosis. Briefly, macrophages were double-stained with anti-mouse CD16-APC (a membrane stain, from BioLegend) and calcein-AM (a marker of active metabolism, from Sigma) at 1, 5, and 24 h of incubation with CFW-labeled yeasts. The percentage of macrophage viability was calculated as the number of macrophages positive for both fluorescence (calcein-AM and anti-CD16) in an infection assay compared to wells with uninfected macrophages (control). Phagocytosing macrophages were quantified as the number of macrophages positive for calcein, anti-CD16 and CFW fluorescence.

### *G. mellonella* survival assays

Infection and killing assays of wax moth larvae were conducted as reported (Pérez-García et al., 2016). Briefly, aliquots containing 2 × 10^7^ yeast cells in 10 μL PBS were passaged through a syringe with a 32-gauge needle, and injected directly into the hemocoel, through the last left pro-leg of the larva, using a Hamilton syringe and a 26-gauge needle. Larvae were maintained at 25°C after injection and survival was monitored daily. Each experimental group contained 10 larvae. PBS-injected and untreated larvae were included in each experiment as controls.

### *C. guilliermondii* infection model and fungal burden

A non-lethal experimental model of disseminated candidiasis was performed as reported (Ifrim et al., 2014; Pérez-García et al., 2016). Animal groups containing five 8- to 12-weeks-old male Balb/c WT mice (22–27 g of weight) were injected via the lateral tail vein with 2 × 10^7^ *C. guilliermondii* yeast cells, previously passaged through a syringe with a 32-gauge needle, in 100 μL of sterile PBS. As a control group, 5 mice were injected with 100 μL of sterile PBS. Animals were mantained with sterile water and normal diet *ad libitum*. Mice were monitored daily and they showed no signs of disease like weight loss, lethargy, ruffled fur or rapid shallow breathing during 5 days following intravenous infection. After 2 or 5 days post infection, animals were *humanitarianly* euthanatized, and the liver, kidneys and spleen were removed, weighed, and separately homogenized with a tissue grinder. The tissue fungal burden was quantified by plating serial dilutions on YPD agar plates. The CFU were counted after 48 h of incubation at 30°C and expressed as CFU/g tissue.

### Statistical analysis

Statistical analysis was performed using GraphPad Prism 6 software. Growth data in the presence of cell wall perturbing agents were analyzed by two-way ANOVA. Cytokine stimulation using human PMBCs was performed in duplicate with six healthy donors, whereas the rest of the *in vitro* experiments were performed at least thrice in duplicate. Data represent cumulative results of all experiments performed. The Mann-Whitney *U* test or unpaired *t*-test was used to establish statistical significance (see figure legends for details), with a significance level set at *P* < 0.05. Experiments with *G. mellonella* were performed three times, with a total of 30 larvae per group (10 larvae for each experiment). Results were analyzed using the Log-rank test and arranged in survival curves using Kaplan-Meier charts. The statistical significance was set at *P* < 0.05.

## Author contributions

MN, NP, and HM conceived the study. MN, TD, KD, KC, EM, AD, RG, VC, MC, NH, LP, DS, and CV performed experiments. MN, TD, KD, CV, AG, RA, TN, ML, NP, and HM analyzed data. HM and NP drafted the manuscript. MN, TD, KD, KC, EM, AD, RG, VC, MC, LP, DS, CV, AG, RA, TN, ML, NP, and HM revised and approved the manuscript. NP and HM equally contributed to this work and are corresponding authors.

### Conflict of interest statement

The authors declare that the research was conducted in the absence of any commercial or financial relationships that could be construed as a potential conflict of interest.
