# Supplementary material for: Disruption of Protein Mannosylation Affects Candida guilliermondii Cell Wall, Immune Sensing, and Virulence
Source: Front Microbiol. 2016 Dec 2;7:1951. doi: 10.3389/fmicb.2016.01951 (PMC5133257; doi:10.3389/fmicb.2016.01951)
Supplement: Supplementary file 1 [file DataSheet1.DOCX]

Supplementary Material

**Disruption of Protein Mannosylation Affects *Candida guilliermondii* cell wall, Immune Sensing and Virulence**

**María J. Navarro-Arias^1^, Tatiana A. Defosse^2, 3^, Karine Dementhon^4^, Katalin Csonka^5^, Erika Mellado-Mojica^6^, Aline Dias Valério^7^, Roberto J. González-Hernández^1^, Vincent Courdavault^2^, Marc Clastre^2^, Nahúm V. Hernández^1^, Luis A. Pérez-García^1^, Dhirendra K Sigh^5^, Csaba Vizler^8^, Attila Gácser^5^, Ricardo S. Almeida^7^, Thierry Noël^4^, Mercedes G. López^6^, Nicolas Papon^3;^*, and Héctor M. Mora-Montes^1;^***

^1^Departamento de Biología, División de Ciencias Naturales y Exactas, Campus Guanajuato, Universidad de Guanajuato, Noria Alta s/n, col. Noria Alta, C.P. 36050, Guanajuato, Gto., México

^2^Université François-Rabelais de Tours, Biomolécules et Biotechnologies Végétales, EA 2106, Tours, France.

^3^Université d'Angers, Groupe d'Etude des Interactions Hôte-Pathogène, EA 3142, Angers, France.

^4^Université Bordeaux 2, Laboratoire de Microbiologie Fondamentale et Pathogénicité, UMR-CNRS 5234, Bordeaux, France.

^5^Department of Microbiology, University of Szeged, Közép fasor 52, H-6726 Szeged, Hungary.

^6^Centro de Investigaciones y de Estudios Avanzados del IPN, Apartado Postal 629, 36500 Irapuato, Guanajuato, México.

^7^Departamento de Microbiologia, Centro de Ciências Biológicas, Universidade Estadual de Londrina, Londrina, PR, Brazil.

^8^Institute of Biochemistry, Biological Research Center of the Hungarian Academy of Sciences, Szeged, Hungary.

***Correspondence:** Nicolas Papon, Tel (+33) 024-4688363, e-mail: [nicolas.papon@univ-angers.fr](mailto:nicolas.papon@univ-angers.fr); Hector M. Mora-Montes, Tel. (+52) 473-7320006 Ext. 8154, Fax (+52) 473-7320006 Ext. 8153, e-mail: hmora@ugto.mx

# Supplementary Data


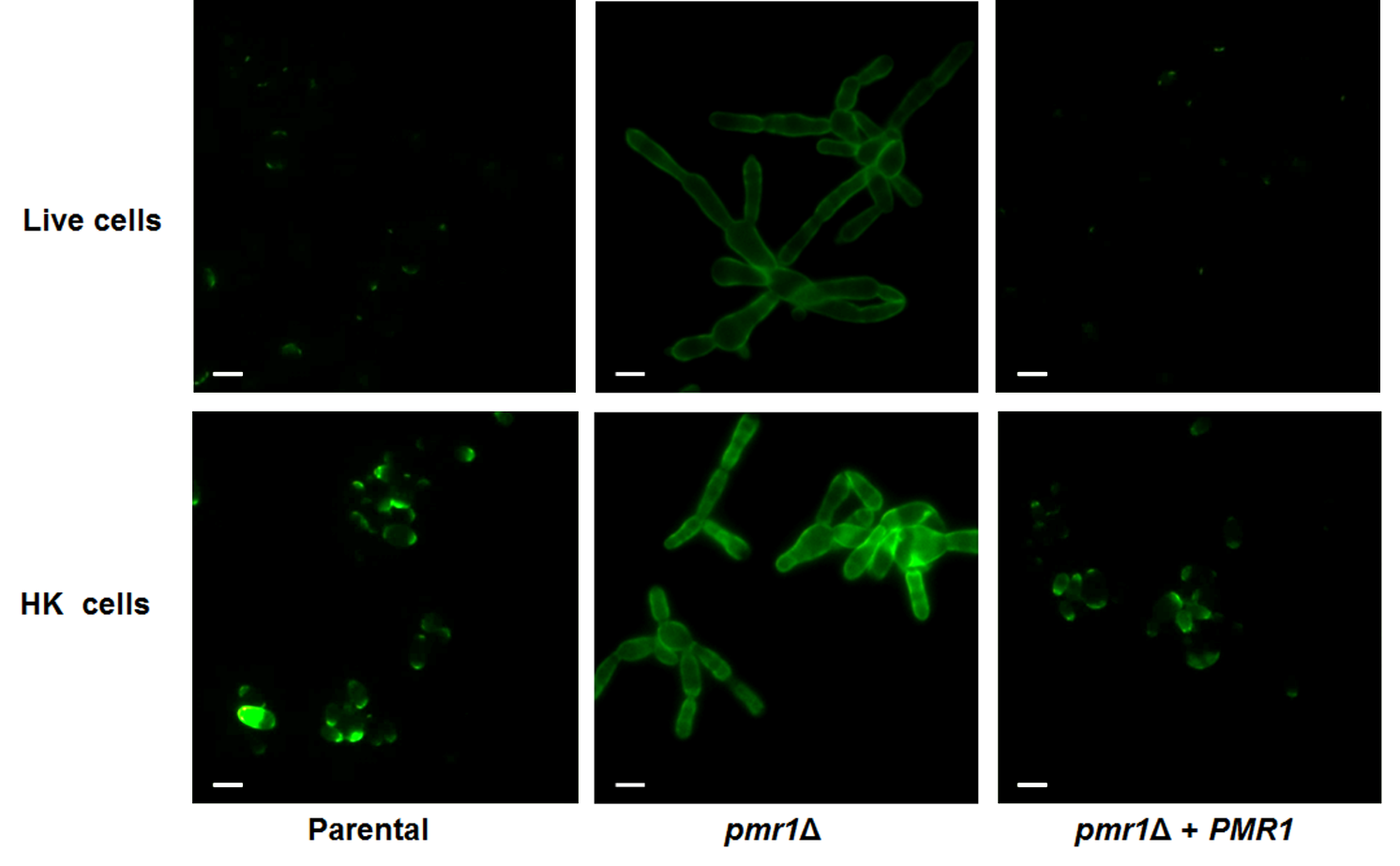


**Figure 1S: The cell-wall structural polysaccharide chitin is significantly exposed at the cell surface of the *C. guilliermondii* *pmr1*Δ null mutant.** Live or heat-killed (HK) cells were incubated with WGA-FITC as described in the Experimental procedures, and inspected under fluorescence microscopy. The strains used are KU141 (Parental), HMY134 (*pmr1*Δ) and HMY138 (*pmr1*Δ + *PMR1*). Scale bar = 10 μm. In live parental and re-integrant control cells, there is labelling only at the budding scars, whereas in HK control cells the lectin also binds to other areas of the cell wall.


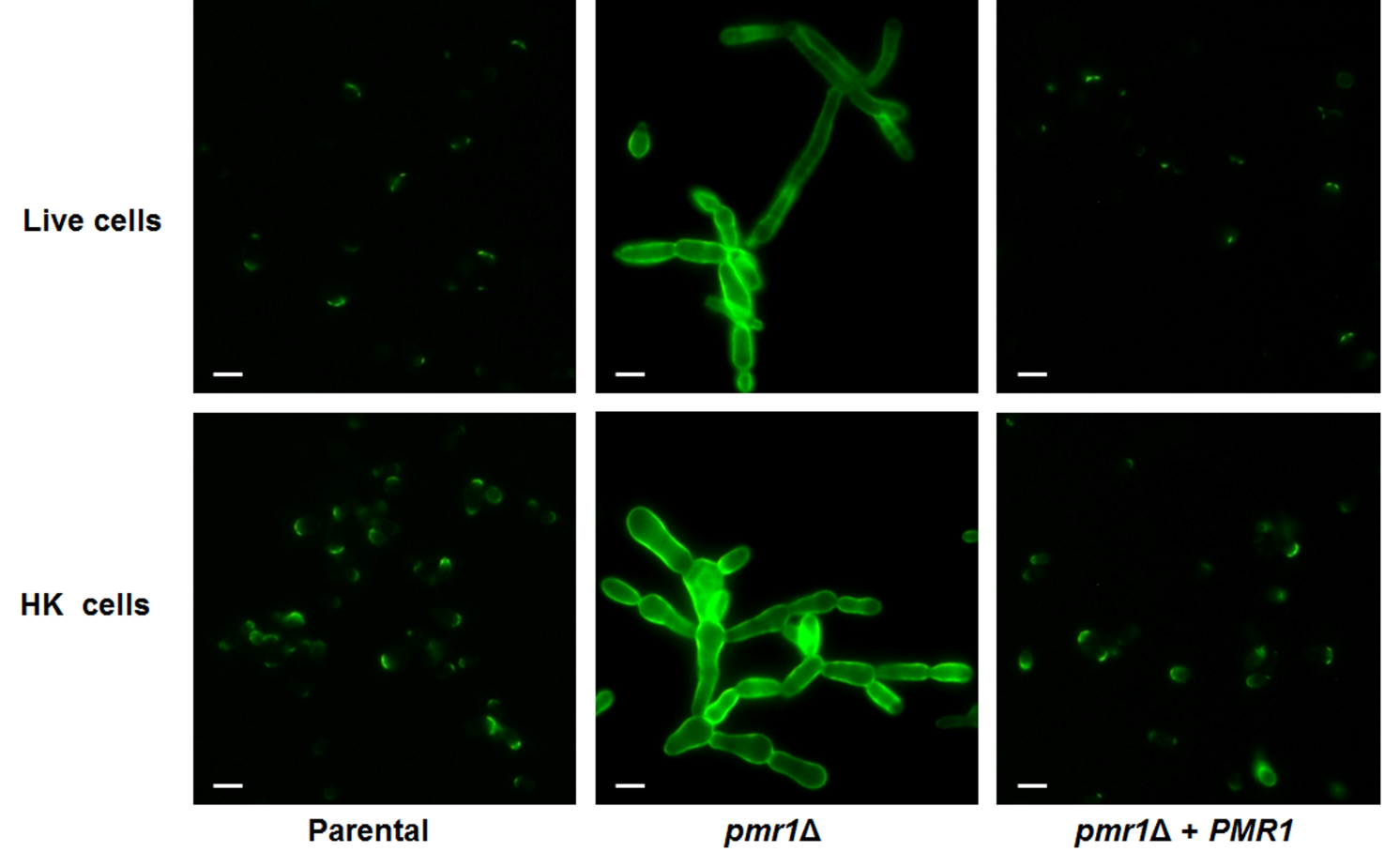


**Figure 2S: The cell-wall structural polysaccharide β1,3-glucan is significantly exposed at the cell surface of the *C. guilliermondii* *pmr1*Δ null mutant.** Live or heat-killed (HK) cells were incubated with IgG Fc-Dectin-1 chimera as described in the Experimental procedures, and inspected under fluorescence microscopy. The strains used are KU141 (Parental), HMY134 (*pmr1*Δ) and HMY138 (*pmr1*Δ + *PMR1*). Scale bar = 10 μm. In live parental and re-integrant control cells, there is labelling only at the budding scars, whereas in HK control cells the lectin also binds to other areas of the cell wall.





**Figure 3S: Blocking of dectin-1 affects cytokine stimulation by *C. guilliermondii* cells.** Human PBMCs were pre-incubated with laminarin 1h at 37°C, before incubation with yeast cells. After 24 h incubation at 37°C the supernatants were saved and used to quantify cytokine levels. Results (means ± SD) where obtained using samples from six donors, each assayed in duplicate wells. The strains used are ATCC 6260 and HMY138 (*pmr1*Δ + *PMR1*). **P* < 0.05, when compared to same cell type without treatment.


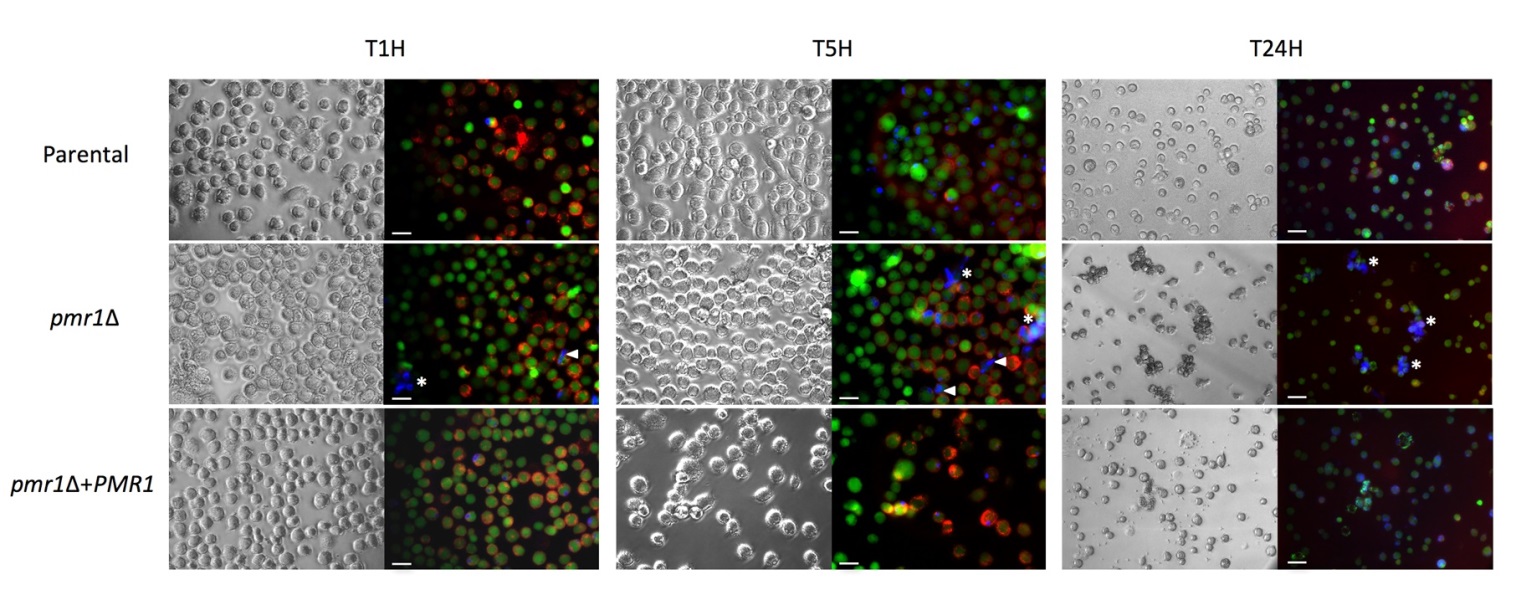


**Figure 4S: Analysis of murine macrophages phagocytosing *C. guilliermondii* cells.** Macrophages were infected with either *C. guilliermondii* KU141 (Parental), HMY134 (*pmr1*Δ), and HMY138 (*pmr1*Δ+*PMR1*) and observed with the microscope after 1 h, 5 h and 24 h of infection. Yeast cells were stained with CFW, macrophages were double-stained with calcein-AM and anti-mouse CD16-APC. Left panel: phase contrast, right panel: fluorescence. The scale bar represents 25 μm. Arrowheads indicate pseudohyphae, * indicate cell-cluster.
